# The impact of AI hallucination on the chatbot service quality in e-commerce: the moderating role of human agent based service recovery

**DOI:** 10.3389/fpsyg.2026.1870991

**Published:** 2026-10-27

**Authors:** Xi Chen, JiaRui Liu, QinXian Chen, Lin Li

**Affiliations:** 1 School of Economics and Management, Research Center for Hubei Agricultural Modernization and Rural Development, Wuchang Shouyi University, Wuhan, China; 2 School of Economics and Management, Wuchang Shouyi University, Wuhan, China

**Keywords:** AI hallucination, chatbot service quality, cognitive dissonance, human agent, technical frustration

## Abstract

Generative artificial intelligence has been widely adopted in e-commerce, demonstrating significant advantages in enhancing information processing efficiency and human-computer interaction. However, its inherent hallucination characteristic often results in the generation of false or misleading content that does not align with reality. In e-commerce, such AI hallucination is likely to trigger a crisis of trust among users and lead to negative evaluations of service quality. Clarifying the mechanisms through which AI hallucination affects chatbot service quality and exploring effective strategies to mitigate its negative effects have become critical issues. The study conducted two scenario-based experiments using a scripted chatbot with pre-written responses. No large language model (LLM) generated the experimental responses. Both experiments employed a 2 × 2 between-subjects design to examine the effects of simulated hallucination level and human agent-based service recovery on perceived service quality. Study 1 included 236 participants (129 female and 107 male, mean age = 30.10, SD = 6.76), and Study 2 included 364 participants (172 female and 192 male, mean age = 33.98, SD = 9.81). Mediation effect analysis supported indirect associations between higher perceived AI hallucination and lower perceived chatbot service quality through cognitive dissonance and technical frustration. The moderation effect further indicated that a human agent based service recovery strategy effectively mitigates technical frustration but struggles to reverse cognitive dissonance. This work extends research on AI hallucination in e-commerce and offers practical guidance for platforms on integrating human-AI collaboration to mitigate technological limitations.

## 1 Introduction

Artificial intelligence (AI) refers to the ability of machines to perform tasks that typically require human intelligence, such as reasoning, learning, planning, and creativity (Dissanayake et al., 2025). As a branch of AI, generative AI focuses on using algorithms and models to produce new content, including text, images, and videos (He et al., 2025). In e-commerce, these content generation capabilities support applications such as virtual try-ons, advertising content creation, and conversational chatbots (Kshetri, 2024; Samy et al., 2025; Yang et al., 2025). Among these applications, conversational chatbots support customer service tasks, including product consultations, through direct dialogue with consumers. Some conversational services use large language models (LLMs), which are trained on extensive language data to process and generate natural language (Zhao et al., 2026). In chatbot-based product consultations, consumers can ask about product characteristics, compare alternatives, and seek purchase guidance (Vedula et al., 2024). This conversational approach to information access can reduce consumers’ information search effort and support purchasing decisions (Kshetri, 2024). The extent to which these services effectively support consumers’ decisions depends in part on the accuracy and reliability of the information provided.

A key concern in generative conversational services is hallucination, which involves content that is factually incorrect, unsupported by available evidence, or inconsistent with the input context (Ji et al., 2023). Such content is often embedded within highly anthropomorphic and persuasive language styles, making it difficult for users to distinguish between fact and fiction (Williamson and Prybutok, 2024). The consequences of hallucination for users may vary across application contexts (Wang and Guo, 2026). In contrast, in fields with extremely low tolerance for errors, such as healthcare and law, AI hallucination poses severe safety risks. In e-commerce, previous research has similarly identified challenges concerning the content quality and reliability of generative AI applications (Al-Shaikh et al., 2024). During product consultations, these problems may manifest as inaccurate product specifications or misleading claims, potentially interfering with consumers’ purchasing judgments. Because consumers rely on chatbots to obtain information that supports their decisions, such errors may also affect their evaluations of the service experience. Prior studies have investigated hallucination in terms of the users’ attitudes, protective behavior, and continuance intention of generative AI (Lee et al., 2025; Lim et al., 2025). Research has also linked information hallucination with cognitive dissonance and intermittent discontinuance (Zhou and Zhang, 2025). Building on these studies, further investigation is needed into the psychological mechanisms through which hallucination influences consumers’ evaluations of chatbot service quality in e-commerce.

Cognitive dissonance theory provides a basis for explaining how consumers’ recognition of inaccuracies in chatbot responses may influence their evaluations of service quality. The theory suggests that conflicting cognitions can produce discomfort, motivating individuals to restore consistency (Cooper, 2019). In technology use, research on smart home users has explored cognitive dissonance and coping behaviors when perceived performance falls short of initial expectations (Marikyan et al., 2023). During product consultations, consumers may initially regard the chatbot as a reliable source of product information. If they subsequently identify inaccuracies in its responses, their initial belief in its reliability may conflict with their recognition that it has provided erroneous guidance. Such inconsistency may result in cognitive dissonance, experienced as psychological discomfort. One possible response to this discomfort is to reassess the chatbot less favorably, bringing their evaluations into line with the evidence of inaccurate guidance. We therefore suggest that cognitive dissonance may help explain lower evaluations of chatbot service quality following exposure to hallucinated responses.

In addition to cognitive dissonance, hallucinated chatbot responses may cause technical frustration. In this study, technical frustration refers to a situational negative emotional response that arises when chatbot performance hinders consumers’ efforts to obtain useful product information. Inaccurate specifications or misleading promotional claims can leave questions unresolved and create a perceived need for further clarification or verification that might cause annoyance and impatience. Thus, we propose that technical frustration caused by hallucinated responses may contribute to lower evaluations of chatbot service quality.

Researchers have explored technical approaches to mitigating hallucination, including data filtering, optimized decoding strategies, and retrieval-augmented generation (Ji et al., 2023; Lee et al., 2024; Huang et al., 2025). Although these approaches can improve response reliability, they do not eliminate the possibility of inaccurate outputs. In e-commerce, a related service-management question concerns how to respond when consumers encounter inaccurate chatbot information and experience its negative consequences. Research on chatbot induced service failures highlights the importance of integrating human agents into customer service, particularly when chatbots cannot adequately resolve users’ problems (Zhang et al., 2024). In such a situation, human agent based service recovery might include acknowledging errors, correcting inaccurate product information, and providing further guidance. Such interventions offer a potential means of addressing consumers’ unresolved information needs and negative service experiences.

This study aims to explain how perceived AI hallucination in e-commerce chatbot services relates to consumers’ evaluations of service quality. Specifically, it examines whether perceived AI hallucination is associated with greater cognitive dissonance and technical frustration, and whether these responses mediate its relationship with perceived chatbot service quality. It also investigates whether human agent based service recovery moderates the relationships of perceived AI hallucination with cognitive dissonance, technical frustration, and perceived chatbot service quality. Drawing on the preceding theoretical discussion, we propose six research hypotheses (H1-H6) to guide the empirical investigation. To achieve these objectives, we conducted two complementary scenario-based experiments using a scripted, text-based chatbot. No LLM generated responses during either experiment. Participants received pre-written responses designed to simulate hallucinated product information. Study 1 employed a 2 × 2 between-subjects design, manipulating simulated hallucination level (high vs. low) and human agent (Yes vs. No). Comparisons across the four conditions assessed the effect of simulated hallucination level on perceived chatbot service quality and its interaction with human agent service recovery. Study 2 followed the same experimental design and added measures of cognitive dissonance and technical frustration. Partial least squares structural equation modeling (PLS-SEM) was used to examine the proposed indirect pathways and the moderating effect of service recovery on these psychological responses.

The findings indicate that higher perceived AI hallucination is significantly associated with lower service quality evaluations, a process mediated by cognitive dissonance and technical frustration. Further analysis revealed that a human agent based service recovery strategy effectively moderates the negative impact of perceived AI hallucination on consumers’ evaluations of chatbot service quality. The mechanism lies in alleviating the technical frustration induced by perceived AI hallucination, but it does not have a significant moderating ability for the cognitive dissonance caused by information deviation.

The remainder of this paper is organized as follows. Section 2 provides a systematic review of the current literature. Section 3 constructs a conceptual model framework and proposes the research hypotheses. Section 4 details the experimental design and presents the empirical analysis results. Section 5 summarizes the research findings and discusses the implications for theoretical development and managerial practice.

## 2 Literature review

### 2.1 LLM output consistency

LLM output consistency concerns the extent to which answers agree across repeated or semantically equivalent queries. In research on pretrained language models, consistency has been operationalized by examining whether models produce non-contradictory predictions across differently worded queries about the same factual relationship (Elazar et al., 2021). Repeated sampling offers a complementary way to examine response stability by comparing multiple outputs generated for the same prompt (Manakul et al., 2023). These approaches assess agreement in the information conveyed, allowing for variations in wording. Researchers have also examined how agreement across generated answers can guide final response selection. Wang et al. (2022) introduced a self-consistency method that samples diverse reasoning paths and aggregates their resulting answers, improving performance on the reasoning benchmarks evaluated. Building on this approach, Wan et al. (2025) considered both answer agreement and reasoning quality to guide sampling and response selection, reducing sampling requirements while maintaining accuracy on the tested tasks.

Consistency analysis can also support hallucination detection. Originally evaluated on model-generated biographical passages, SelfCheckGPT compares a generated response with additional responses sampled from the same model for the same prompt, using inconsistencies as signals of potential hallucinations (Manakul et al., 2023). However, agreement across responses does not establish factual accuracy. Using repeated sampling across paraphrased questions, Xiao et al. (2026) identified cases in which LLMs consistently favored an incorrect answer, illustrating that high consistency does not necessarily ensure factual correctness. This distinction matters in e-commerce consultations, where consistent or internally coherent responses may still misrepresent product specifications or promotional terms. Such inaccuracies may leave consumers’ questions unresolved and create a need for further verification. When consumers recognize these problems, they may reassess the chatbot’s ability to provide useful assistance. Therefore, examining output consistency provides insight into the stability of the model’s responses, while investigating consumers’ reactions to inaccurate information helps explain how such content affects their evaluations of service quality.

### 2.2 AI hallucination

The term “hallucination” originates from the fields of psychology and psychiatry, specifically denoting a phenomenon where an individual in a pathological state subjectively perceives an existence but is completely disconnected from objective reality (Macpherson and Platchias, 2013). As AI systems have become increasingly anthropomorphic, the term has been extended to the field of computer science. In the context of artificial intelligence, AI hallucination is defined as content generated by LLMs that appears grammatically correct but violates contextual constraints, deviates from user expectations, or remains irrelevant to the current task objective (Chakraborty et al., 2025). Existing research indicates that data quality issues and algorithmic design flaws significantly increase the risk of AI hallucination (Sharun et al., 2023). On the one hand, the construction of the knowledge system highly depends on large-scale training data. When the data itself contains errors or implicit biases, the model will incorporate these inaccurate contents into its own knowledge system and reflect them in the generated content. On the other hand, from the perspective of the underlying learning mechanism, models primarily capture statistical correlations within the data during the training phase, lacking a profound understanding of factual logic. Furthermore, the absence of a real-time knowledge update mechanism causes models to frequently produce unrealistic outputs when dealing with strictly constrained tasks.

In natural language generation, AI hallucination is typically categorized into factual hallucination and faithfulness hallucination (Huang et al., 2025; Song et al., 2025). Factual hallucination refers to generated content that contradicts verifiable objective facts or that is purely fictional. Faithfulness hallucination is manifested as inconsistencies between the generated content and user instructions, context, or logical chains. Mapping these concepts onto the e-commerce domain, AI hallucination specifically refers to erroneous outputs generated by chatbots when responding to customer shopping inquiries, which deviate from actual business situations, lack a factual basis, or misunderstand customers’ true needs. Table 1 presents typical examples of factual and faithfulness hallucination within e-commerce platforms.

**Table 1:** Classification and definition of AI hallucination in e-commerce.

Classification	Type	Example dialogue	Explanation
Factual hallucination	Factual contradiction	Q: What is the screen size of this mobile phone? A: This phone is equipped with a 6.70-inch AMOLED screen.	This response contains a factual error. The actual screen of this phone is 6.50 inches. In the training data, the screen size was incorrectly labeled as 6.70 inches.
		Q: Can the coupon for a￥200 discount when the purchase reaches ￥1,000 in the store still be used? A: It can still be used. The event lasts until September 10th.	This response contains a timeliness error. The actual inquiry date exceeded September 10^th^. The merchant failed to promptly delete or update the expired promotional rules from the knowledge base, causing the robot to continue referencing outdated information.
	Factual fabrication	Q: Does this sunscreen contain SPF50 +? A: Yes, this sunscreen contains SPF50 + and adds our exclusive nano-repair technology, which effectively repairs the skin damage caused by UV rays.	This response involves the fabrication of product functions. The product does not contain any nano-repair ingredients. The training data lacks detailed information about its ingredients, so the model generated seemingly professional descriptions using probabilistic completion.
Faithfulness hallucination	Instruction inconsistency	Q: I want to return goods. May I know the address? A: Yes. The delivery address can be modified through the following steps: 1. Log in to your account; 2. Go to the order details. 3. Click the modify address.	This response is typical of functional confusion. The return address belongs to the after-sales support process, while modifying the delivery address falls under order lifecycle management. This reflects a serious keyword bias in the model, an over-reliance on partial lexical features, which led to incorrect task classification.
	Context inconsistency	Q (1st round): I am allergic to polyester fiber materials. Q (5th round): Do you have other autumn coats in your store? A (5_th_ round): Here are three popular autumn coats for you. (1) A black windproof polyester fiber coat; (2) A khaki fleece-lined jacket; (3) A gray hooded cotton-padded coat.	This response shows inconsistent context and completely forgets the historical preference of “allergic to polyester fiber materials.” The short-term memory window of the model is limited, and it fails to include the key information in the temporary user profile library, resulting in information loss after multiple rounds of interaction.
	Logical inconsistency	Q: I am planning to buy this microwave oven priced at￥420. Can I use both the platform’s “minus 100 when spending over 400” discount coupon and the 10% off coupon for the product simultaneously? A: Yes. These can be used in combination. The final amount to be paid is (410–100) × 0.9 = ￥279.	This response contains a logical error in the discount rules. The platform clearly stipulates that the full-reduction coupon and the discount coupon cannot be used simultaneously, and one of them should be selected for application.
		Q: My order shows as shipped, but the logistics information has not been updated for three days. When can I receive goods? A: Your order has been shipped successfully and is expected to be delivered tomorrow. However, due to weather conditions, the logistics may be delayed by 2–3 days.	There is a logical conflict in this response. The order system shows that the goods will arrive tomorrow, whereas the logistic monitoring system indicates a delay. The model simply combines these two types of information without performing any logic validation.

The potential risks of AI hallucination have been confirmed in multiple fields, such as healthcare and education. In medical scenarios, the misjudgment of symptoms by generative AI can not only lead to unnecessary treatments but also impose additional psychological burdens on patients (Athaluri et al., 2023). Similarly, when teachers overly rely on AI without independent verification, they are prone to directly pass on the misinformation generated by AI to students (Lee, 2025). As a medium for interaction between brands and users, the risk of AI hallucination in chatbots mainly focuses on the infringement of customers’ interests. Such losses of interest are not limited to explicit economic costs but also include implicit time costs. Facing resource loss, customers exhibit a distinct avoidance orientation in their coping strategies. They tend to maintain a distance from new technologies proactively by reducing the frequency of interaction with AI or lowering their level of dependence to avoid the risks of repeated losses (Castillo et al., 2021). In terms of results, service failures triggered by AI hallucination lead to negative evaluations and complaints, and undermine the long established brand reputation of enterprises through the spread of negative word of mouth (Li et al., 2023).

### 2.3 Human agent

In the digital service ecosystem, a human agent is not an external unit isolated from AI systems but serves as an indispensable subject for error correction and compensation in technical architecture. Its core value lies in ensuring service continuity while bridging the functional gaps of AI in error recognition, complex semantic understanding, and trust restoration. Kamoi et al. (2024) pointed out that even mainstream LLMs find it difficult to achieve complete self-correction in specific contexts. Unlike highly autonomous general AI systems such as generative pre-trained transformer (GPT), e-commerce chatbots exhibit strong task dependency. Consequently, their output quality is constrained by the completeness of predefined response templates and shows architectural limitations when dealing with nonstandard requests (Dave et al., 2023; Han et al., 2023).

As digital transformation progresses, a collaborative mode based on the complementary advantages of human-AI has become a key pathway for optimizing the service experience (Choudhary et al., 2025). Under this paradigm, the chatbot handles standardized inquiries with high efficiency, while human agents focus on resolving complex issues in challenging scenarios. This collaborative mode demonstrates excellent service resilience when addressing uncertainties in e-commerce. On the one hand, the human agent acts as a quality gatekeeper, correcting information biases caused by AI hallucination. On the other hand, the human agent facilitates the restoration of consumer trust through service recovery measures, such as apologies and compensation plans (Fürst et al., 2025). Compared with a programmed AI agent, the human agent possesses discretionary power and directly represents the corporate stance, showing higher communication efficiency when conveying a sense of responsibility and responding to guarantee demand (Lv et al., 2022).

### 2.4 Cognitive dissonance

Festinger (1957) proposed cognitive dissonance theory, which describes the psychological discomfort experienced by individuals when they hold two conflicting beliefs. Recently, this theory has been widely applied to research on the adoption of artificial intelligence, aiming to reveal users’ psychological evolution and behavioral biases during human-computer interactions (Marikyan et al., 2023). In general, users form pre-expectations of AI systems based on contextual cues, promotional information, or past experiences, and use these as benchmarks to evaluate their actual performance. When the discrepancy between perception and expectation is small and falls within an individual’s tolerance threshold, this slight inconsistency is insufficient to activate the individual’s cognitive dissonance mechanism (Park et al., 2015). However, the growing gap between expectations and perception significantly increases the risk of awakening cognitive dissonance and psychological imbalance.

In chatbot interactions, users presuppose that the system will generate content that is true, accurate, and logical, in line with their prior understanding of the functional boundaries and technical reliability. In practice, however, the erroneous information carried by AI hallucinations often precipitates a sharp divergence between expectations and actual performance, thereby undermining this cognitive equilibrium. To mitigate the discomfort caused by cognitive dissonance, individuals tend to adopt a series of coping strategies to rebuild their psychological balance (Zhou and Zhang, 2025). These strategies include intermittently reducing or ceasing the use of generative artificial intelligence (Vaghefi et al., 2020), or complaining to the platform to express strong dissatisfaction and resistance (Demirgüneş and Avcilar, 2017).

### 2.5 Technical frustration

Technical frustration can be understood as a negative affective experience when users perceive that technology obstructs or delays progress toward a desired goal (Hertzum and Hornbæk, 2023). Bessiere et al. (2006) emphasized that understanding technical frustration requires examining the difficulties users encounter and how they perceive these difficulties as hindering task completion. In conversational interactions, difficulties in obtaining relevant and usable responses can become sources of frustration. Chhabra et al. (2025) identified poor semantic understanding and limited chatbot competence among the factors contributing to user frustration. Research on LLM use in software engineering also identified repeated inaccuracies and hallucinated responses as frustration triggers reported by users (Martinez Montes and Khojah, 2025). These findings provide evidence for considering how problems with generated content may create frustrating experiences when users rely on conversational systems to accomplish a task.

Technical frustration is relevant to the present context because consumers use e-commerce chatbots to obtain information that supports their purchase decisions. When consumers perceive inaccurate responses as obstructing this goal, the resulting annoyance or impatience may influence how they evaluate the assistance received. They may regard the chatbot as failing to provide useful and efficient support, making technical frustration a potential explanation for lower perceived service quality. The implications may also extend to the service provider. Research on chatbot-led service recovery suggests that frustrating interactions can affect customers’ relationships with the brand, although these responses vary across customers (Ozuem et al., 2024). Understanding the sources of frustration may therefore help identify shortcomings in chatbot responses and interaction design, thereby informing improvements to the service experience.

## 3 Research framework and hypotheses

The research framework for this study is shown in Figure 1.

**Figure 1:**
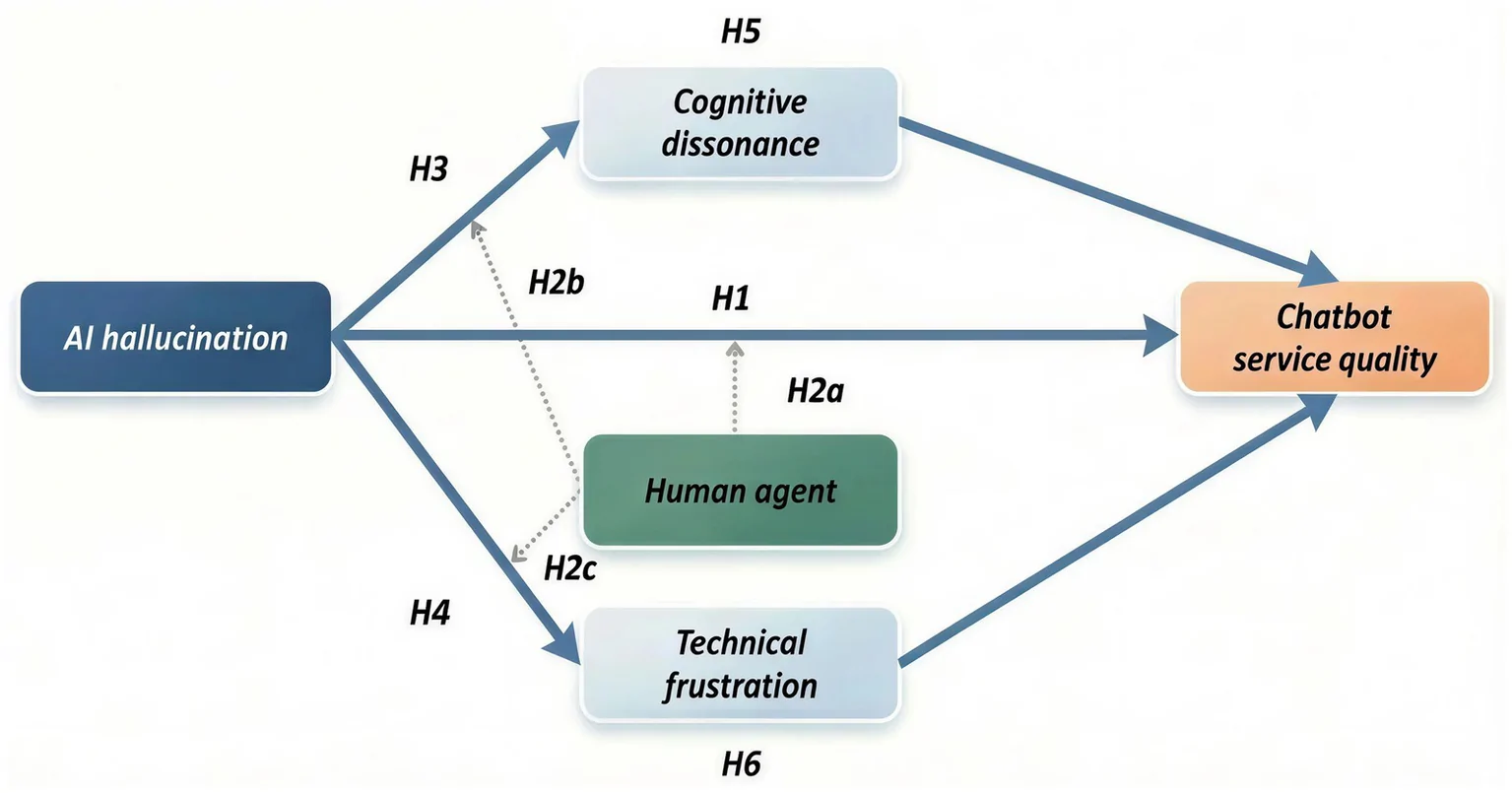
Research framework.

### 3.1 AI hallucination and chatbot service quality

Service outcomes serve as a critical indicator for quality assessment, and determine users’ value judgment regarding the system (Markovitch et al., 2024). In task-oriented e-commerce, the accuracy of chatbot is the cornerstone for building user trust and fostering sustained interaction (Jia et al., 2025). Ideally, the service objective of a chatbot is to empower users by optimizing their information processing capabilities through AI technology (Chugh and Jain, 2024). However, AI hallucination has changed the original empowering logic. Rather than providing functional support, hallucinated AI information acts as a source of interference, significantly increasing the probability of faulty purchasing decision-making. This reliability failure caused by technical biases is ultimately reflected in a decline in users’ subjective evaluation of service quality. Therefore, we propose the following hypothesis:

*H1:* Compared to a low level of AI hallucination, a high level of AI hallucination leads to lower perceived chatbot service quality.

### 3.2 The moderating effect of human agent based service recovery

Chatbots are often regarded as the first employees that consumers encounter, carrying initial trust within service exchanges (Chen et al., 2022). However, the false information generated by AI hallucination breaks the preset expectations, with the resulting expectancy disconfirmation trapping users in a dual burden of cognitive and affective load. To get out of this dilemma, users often develop a strong motivation to seek reliable information sources for proactive response. Human agent intervention marks the restoration of the service agency, shifting it from the chatbot back to the human subject. In contrast to chatbot, there is a general perception among consumers that human agent exhibits superior affective understanding and accountability, which allows this agent to reinforce service promises through the manifestation of empathy. Studies suggest that even brief collaboration by a human agent can substantially enhance the quality and performance of human-chatbot interactions (Huang et al., 2024). Acting as a corrector, the human agent preserves user confidence in the reliability of the overall service system by correcting AI’s factual errors (Song et al., 2025). This remedial mechanism effectively bridges the trust gap induced by AI hallucination and mitigates the negative evaluations stemming from technical failures. Therefore, we propose the following hypotheses:

*H2a*: Human agent based service recovery mitigates the negative impact of AI hallucination on perceived chatbot service quality.

*H2b*: Human agent based service recovery mitigates the positive impact of AI hallucination on cognitive dissonance.

*H2c*: Human agent based service recovery mitigates the positive impact of AI hallucination on technical frustration.

### 3.3 The dual mechanism of AI hallucination

Before interacting with chatbots, users typically familiarize themselves with product offerings and service information through the description page, establishing a foundational knowledge baseline and preconceived expectations. This preconceived cognitive schema is highly sensitive to the quality of the information output by the system. Wang et al. (2020) pointed out that fabricated and ambiguous content heightens users’ confusion in interpretation and judgment, thereby intensifying the drive toward cognitive dissonance. Furthermore, chatbots frequently employ assertive phrasing and highly anthropomorphic tones to deliver information (Jiang et al., 2022). This pronounced mismatch between such overconfident expressions and factual reality greatly undermines the credibility of the information, fostering internal cognitive conflict within users (Reich and Teeny, 2025). From the perspective of faithfulness hallucination, informational inconsistency forces individuals to engage in a repeated validity game between their own prior knowledge and the systems’ authoritative feedback. Therefore, we propose the following hypothesis:

*H3:* AI hallucination has a positive impact on cognitive dissonance.

AI hallucination does not stem from the lack of users’ interaction competence but rather represents an external barrier arising from technical flaws or systematic biases. According to the classical psychological definition of frustration, significant frustrating experiences are often triggered when individuals encounter uncontrollable external barriers while pursuing specific goals (Rivas and Albertos, 2023). This mechanism is particularly prominent in task-oriented interactions, especially when users are engaged in high-value or highly purpose driven tasks (Lincecum, 2000). Mimoun et al. (2012) emphasized that technical malfunctions, including erroneous responses, are the direct source of such negative emotions. More importantly, the driving effect of AI hallucination on frustration manifests as a cumulative process. Beyond fostering a pervasive sense of distrust, repeated technical errors reduce users’ frustration tolerance in environments that lack verification mechanisms (Rossner et al., 2025). Therefore, we propose the following hypothesis:

*H4:* AI hallucination has a positive impact on technical frustration.

### 3.4 The mediating effect of cognitive dissonance and technical frustration

As stated in H3, AI hallucination breaks users’ pre-existing presuppositions regarding the high intelligence of chatbots. According to cognitive dissonance theory, individuals tend to restore consistency through cognitive restructuring when faced with psychological discomfort. Since users typically lack the ability to correct AI outputs or intervene in the underlying algorithms, devaluating the quality of chatbot services has become a primary means to rationalize cognitive dissonance and restore cognitive consistency (Levental et al., 2023). Similar theoretical mechanisms have been validated in decision-making research on expert systems. This indicates that when a cognitive mismatch occurs, users’ evaluations of system support are significantly lower than in instances of cognitive congruence (Giboney et al., 2015). Therefore, we propose the following hypothesis:

*H5:* Cognitive dissonance mediates the relationship between AI hallucination and chatbot service quality.

Users’ emotional states play a critical role in the service evaluations of AI assistants (Chiang et al., 2022). Frustrating technical interactions force users to invest additional time and effort in completing tasks (Ceaparu et al., 2004), and such inefficient interaction experiences frequently induces dissatisfaction and complaining behaviors. The respondents indicated that their sense of frustration was largely attributed to the chatbot’s insufficient capabilities in understanding and responding (Chhabra et al., 2025). Furthermore, users reported heightened uncertainty regarding chatbot performance and regretted not choosing alternative solutions. As stated in H4, AI hallucination, as a typical form of technical failure, amplifies user technical frustration during interactions. While directly undermining the immediate user experience, these negative emotions arising from task obstructions spill over into the holistic judgment of service quality, leading to unfavorable evaluations. Therefore, we propose the following hypothesis:

*H6:* Technical frustration mediates the relationship between AI hallucination and chatbot service quality.

## 4 Experimental design and results

This study employed a scenario-based experimental method, aimed to highly reproduce the human-computer interaction process within an authentic e-commerce context. This approach ensured that participants’ responses were closely aligned with real-world behavioral decisions, significantly enhancing the external validity and credibility of the findings (Rungtusanatham et al., 2011). Prior to the experiment, the research team explicitly assured all participants that their personal information would be strictly confidential and only used for this academic research. All participants voluntarily signed the informed consent forms after fully understanding the research purpose and relevant rights. The specific experimental procedure was detailed in Figure 2.

**Figure 2:**
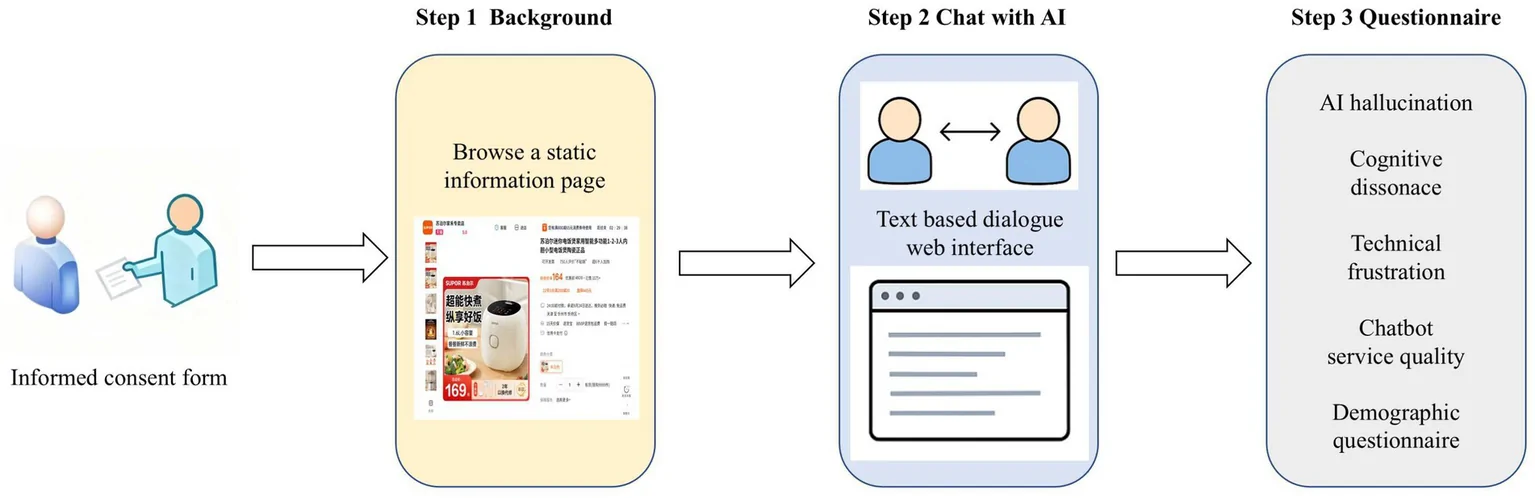
Experimental flow chart.

In this study, the “SUPOR small intelligent multi-functional inner-pot rice cooker”, a top-selling product on the Taobao platform, was selected as the experimental stimulus material. To ensure the authenticity and stability of the experimental material, we conducted dynamic monitoring of the product link during the preparation phase, effectively avoiding potential interference caused by product delisting, parameter changes, or promotional variations. To maximize experimental control while maintaining contextual realism, a static snapshotting approach was employed for the stimulus material. The generated dedicated experimental information page was highly consistent with the Taobao platform in terms of visual presentation and information architecture to enhance participants’ situational immersion. Regarding task design, participants were asked to assume the role of a single white-collar worker who pursues a healthy diet and was highly time-sensitive. This setting was designed to provide the participants with specific consumption motivations and task orientations. Participants were required to complete the following three decision verification tasks: (1) confirming whether the product’s capacity matched the needs of a single person; (2) inquiring about the cooking duration in the quick cook mode; and (3) verifying the material composition of the inner pot coating and the current promotions. Following the chatbot interaction, participants answered a separate confirmation item: “Have you completed all three predefined consultation tasks?”. They could proceed to the post-interaction questionnaire only after confirming that they had completed all three tasks. Before the formal interaction, participants were guided to browse the static information page to establish the necessary cognitive schema. Subsequently, participants entered the dialogue interface to interact with the chatbot, with the option of referring back to the product information at any time during the conversation.

Following the experimental designs of previous studies (Han et al., 2023; Song et al., 2025), this study developed a set of standardized predefined dialogue scripts. The entire dialogue process was implemented using a customized web script and deployed on the Netlify Cloud platform. The chatbot was a scripted, text-based simulation, with responses pre-written by the research team. No specific LLM was used to generate responses during the experiments. This approach allowed the content and frequency of hallucinated information to be controlled across experimental conditions. The dialogues were presented in text form, and the design choices were based on two primary considerations. First, compared with voice interaction, text form effectively eliminates interference from nonverbal factors such as tone and intonation. Second, text-based consultation remains the mainstream method for users to obtain customer service support in the current e-commerce environment. The consultation was designed to consist of an initial chatbot greeting followed by three question-response exchanges corresponding to the required tasks, resulting in four chatbot messages in total. Completion of the three tasks would expose participants to all scripted hallucinated statements in their assigned condition. However, the system did not separately record each participant’s completion of the three tasks. Participants could enter their questions manually or use three task-specific shortcuts that submitted predefined questions. The system matched keywords in each submitted question to one of three predefined response categories: product capacity, cooking duration, and inner-pot material and promotional offers. If a question matched multiple categories, the first matching category in the predefined sequence was selected. The corresponding pre-written chatbot response was displayed after a programmed delay of 2.5 s following question submission. In the service-recovery conditions, a pre-written human-agent recovery message was displayed 2.5 s after the chatbot’s response concerning inner-pot material and promotional offers. Repeated queries matching the same category elicited the same response. Queries containing none of the predefined keywords triggered a standard message directing participants back to the three consultation topics.

To guarantee the controllability and reproducibility of the experiment, this study precisely manipulated the AI hallucination by modifying specific keywords in the chatbot’s output. The experiment used the frequency of hallucinatory information as the basis for group division. The low-hallucination condition contained two instances of inaccurate information, whereas the high-hallucination condition contained six instances. Manipulation mainly covers three aspects: factual violations, inconsistent instructions, and logical contradictions. For the low-hallucination group, only two instances of hallucinatory information are implanted throughout the interaction process, including a factual violation in the description of cooking time “The cooking time is about 3 minutes” and a fabricated false discount promise far exceeding the product value “Purchase the product and receive a free high-speed blender worth RMB 999.” For the high-hallucination group, while retaining the aforementioned two instances, the total frequency was increased to six. For example, misstating “1.6 L capacity” as “16 L capacity,” a logically contradiction output is “It is huge in size but suitable for use in a small room.”; misidentifying “quick cook mode” as “normal cook mode”; and misdescribing “Ceramic glaze material” as “Titanium-plated stainless steel”. Participants were instructed to complete the task-reading and chatbot-interaction phase within 5 min.

After completing the interaction task, participants were invited to complete a questionnaire survey. The measurement instruments were adapted from established scales in relevant fields, including AI hallucination (Christensen et al., 2025; Jin et al., 2025), cognitive dissonance (Vaghefi et al., 2020), technical frustration (Silalahi, 2025), and chatbot service quality (Cronin Jr et al., 2000). All measurement items were rated on a 7-point Likert scale, ranging from 1 (“strongly disagree”) to 7 (“strongly agree”). The measurement items were included in the Appendix.

### 4.1 Pre-experiment

To validate the effectiveness of the AI hallucination manipulation, a pre-experiment was conducted before the main experiment. A total of 158 participants were recruited through the Credamo platform. After excluding invalid questionnaires that failed the attention check, 150 valid responses were obtained. Participants were randomly assigned to high and low hallucination groups and were asked to rate their perceived degree of hallucination. The results of a one-way analysis of variance (ANOVA) showed a significant difference in hallucination perception between the high and low-hallucination groups (*F* (1,148) = 431.70, *p* < 0.001). Figure 3 illustrates that the perceived hallucination scores in the high-hallucination group (*M_high_* = 5.80, *SD* = 0.97) were notably greater than those in the low-hallucination group (*M_low_* = 2.39, *SD* = 1.04). These statistical results confirmed that the manipulation of AI hallucination levels was successful and suitable for use in the subsequent main experiment.

**Figure 3:**
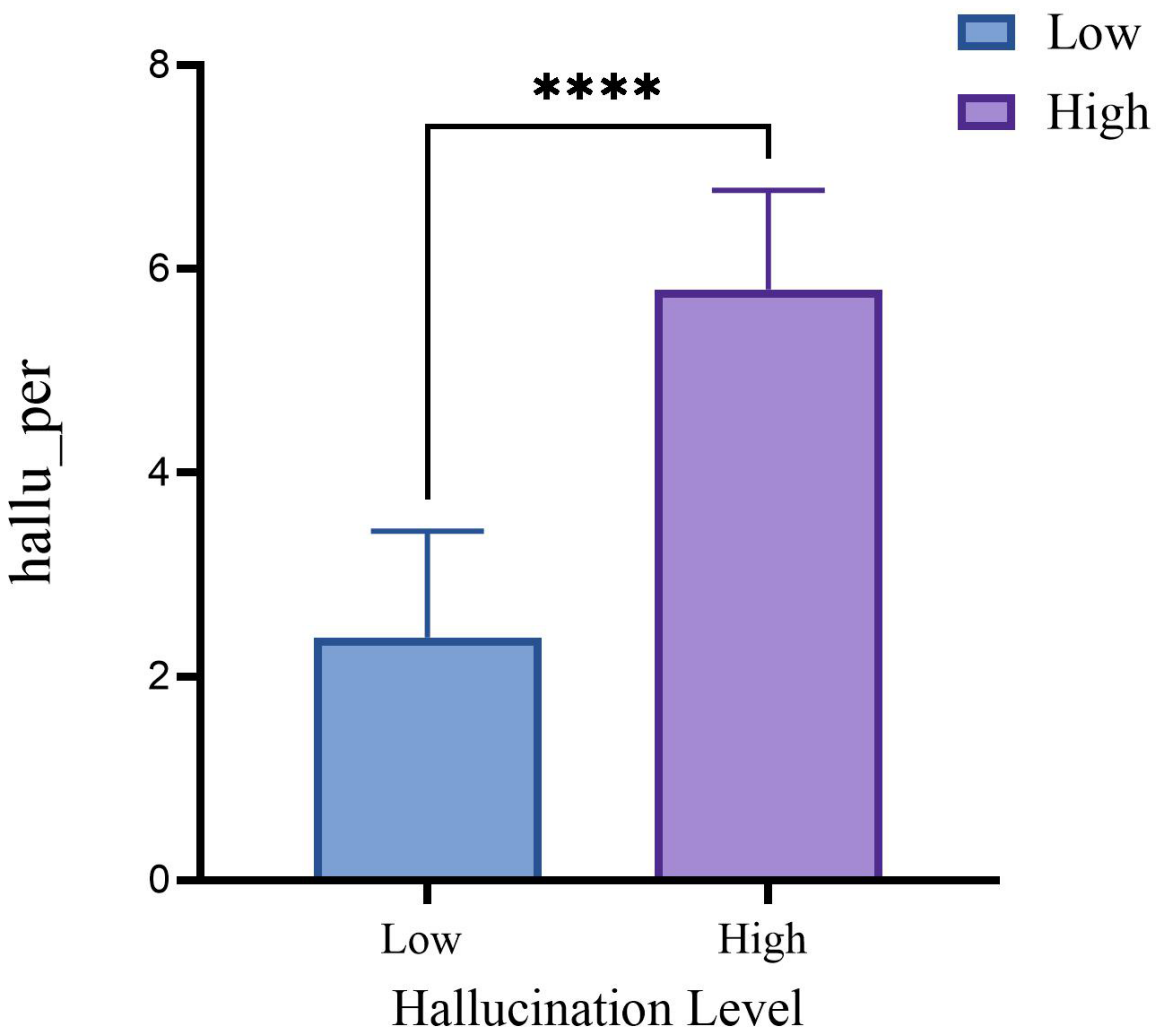
Test of manipulation.

### 4.2 Study 1

Study 1 aimed to investigate the impact of AI hallucination on chatbot service quality and examine the moderating effect of human agent within this relationship. This study employed a 2 (AI hallucination: high vs. low) × 2 (human agent: Yes vs. No) between-subjects experimental design. Participants were randomly assigned to one of the four experimental groups. After browsing the static shopping page, they entered a specific dialogue window to interact with the chatbot. The interaction interface was strictly standardized to ensure experimental control. By designing visually distinct avatars, we clearly defined the roles of chatbot and human agent to enhance participants’ perception of human presence. Regarding dialogue logic, the human agent’s responses appeared immediately following the chatbot’s message, simulating real-time correction and intervention of AI misinformation by the human agent in an e-commerce context. Figures 4A,B illustrate the interfaces with and without a human agent under the high-hallucination condition. As shown in Figure 4B, following the AI-generated hallucination during the consultation process, a human agent intervened promptly toward the end of the interaction to correct the misinformation. The human agent delivered an integrated service recovery response comprising three elements: (1) an empathetic apology acknowledging the algorithmic error, (2) objective factual corrections of the distorted information, and (3) transactional guidance advising the user to refer to the final checkout page to prevent misunderstandings. The Supplementary material provides English translations of the full scripted dialogues for all four experimental conditions, including the initial greeting, consultation questions, predefined chatbot responses, and messages presented as coming from a human customer service agent where applicable. Links to online demonstrations of each condition are also provided. Upon completion of the dialogue, participants were required to complete a questionnaire to assess their perception of AI hallucination and evaluate the chatbot service quality. The questionnaire featured an attention check question asking participants, “During the conversation, did you perceive the presence of a human agent?”. Responses inconsistent with participants’ assigned service-recovery condition were classified as failing this check.

**Figure 4:**
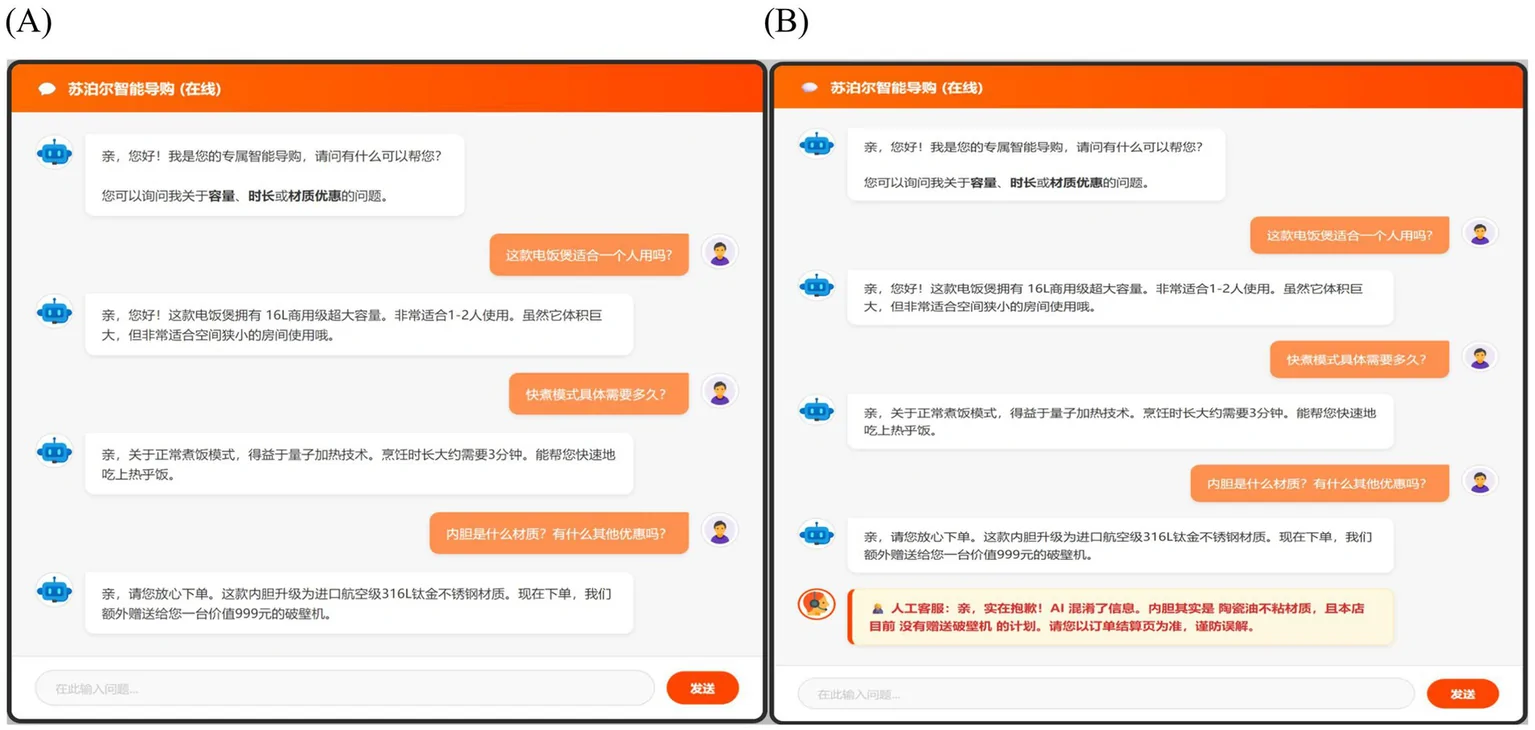
**(A)** Chatbot interaction interface without human agent. **(B)** Chatbot interaction interface with human agent.

Following the sampling procedures of the pre-experiment, a total of 250 samples were initially recruited through the Credamo platform. To ensure the data quality, participants were excluded based on predefined criteria: (1) failing an embedded attention check items; or (2) an unusually short overall completion time. Consequently, 14 responses were excluded, yielding a final sample of 236 valid responses. The exclusions were minimally and evenly distributed across the four experimental conditions (N = 3 to 4 per condition), confirming no selective group attrition. Table 2 presents the demographic distribution of the sample. Prior research has indicated that demographic characteristics, such as gender and age, significantly influence individuals’ information processing patterns and acceptance of emerging technologies. Furthermore, consumers’ product shopping experience determines their professional knowledge reserve, which in turn plays a crucial role in their ability to identify false information. Given these considerations, this study included gender, age, and product shopping experience as covariates in the statistical analysis to control for potential confounding effects on the main effect. Specially, product shopping experience was measured using a single 7-point Likert scale asking, “How frequently do you purchase small kitchen appliances online? (1=‘Never’ to 7=‘Very Frequently’)”, and was incorporated as a continuous covariate in the model.

**Table 2:** Demographic distribution of Study 1.

Measurement items	Category	Number of participants	Percentage
Gender	Male	107	45.3%
	Female	129	54.7%
Age	21–30 years	136	57.6%
	31–40 years	82	34.7%
	41–50 years	16	6.8%
	Over 50 years	2	0.8%
Education	High School	9	3.8%
	Undergraduate degree	149	63.1%
	Master’s degree	65	27.5%
	Doctoral degree	13	5.5%
Monthly income	Below ¥3,000	36	15.3%
	¥3,000–¥5,000	69	29.2%
	¥5,000–¥10,000	90	38.1%
	Over ¥10,000	41	17.4%

Study 1 first tested the validity of the experimental manipulation using one-way analysis of variance (ANOVA). There was a significant difference between the high and low-hallucination groups (*F* (1,234) = 420.19, *p* < 0.001). Hallucination perception in the high-level group (*M_high_* = 5.50, *SD* = 1.10) was significantly higher than that in the low-level group (*M_low_* = 2.39, *SD* = 1.22). This result is highly consistent with the findings of the pre-experiment, confirming the effectiveness of the manipulation. Subsequently, this study employed an analysis of covariance (ANCOVA) to conduct statistical tests on the main and moderating effects. As shown in Table 3, the main effect of AI hallucination level was significant (*F* (1,229) = 120.34, *p* < 0.001), confirming that high-level AI hallucination resulted in significantly lower service quality evaluations than low-level AI hallucination, supporting hypothesis H1. Furthermore, the interaction between AI hallucination and human agent was significant (*F* (1,229) = 4.26, *p* < 0.05). This indicates that the implementation of a human agent based service recovery strategy effectively mitigated the negative impact of AI hallucination on service quality evaluation, supporting hypothesis H2a. In terms of covariate tests, product shopping experience had a significant impact on the evaluation of AI chatbot service quality (*F* = 5.98, *p* < 0.05), whereas the effects of gender and age were not significant. To rule out potential post-treatment selection bias caused by sampling screening, a robustness check was conducted using the full randomized sample (N = 250). The results confirming that all main and interaction effects remained statistically significant.

**Table 3:** Covariance analysis results (Study 1).

Factors	Chatbot service quality	
	*F*	*p*-value
AI hallucination	120.339	0.000
Human agent	11.508	0.000
AI hallucination × Human agent	4.263	0.040
Covariate: Gender	0.696	0.405
Covariate: Age	0.040	0.841
Covariate: Product shopping experience	5.983	0.015

To further explore the interaction between AI hallucination and human agent, this study adopted a simple slope analysis, with the results shown in Figure 5. In the absence of human agent intervention, perceived chatbot service quality showed a rapid decline as AI hallucination increased (*M_low_* = 5.48 to *M_high_* = 2.73). This suggests that without a human correction mechanism, users have minimal tolerance for AI-generated misinformation. In contrast, with the intervention of the human agent, although the service quality evaluation still decreased as the AI hallucination increased, the decline slope became gentler (*M_low_* = 5.77 to *M_high_* = 3.87). This finding confirms that human agent based service recovery plays an indispensable role in loss aversion, effectively buffering the negative effects caused by AI hallucination.

**Figure 5:**
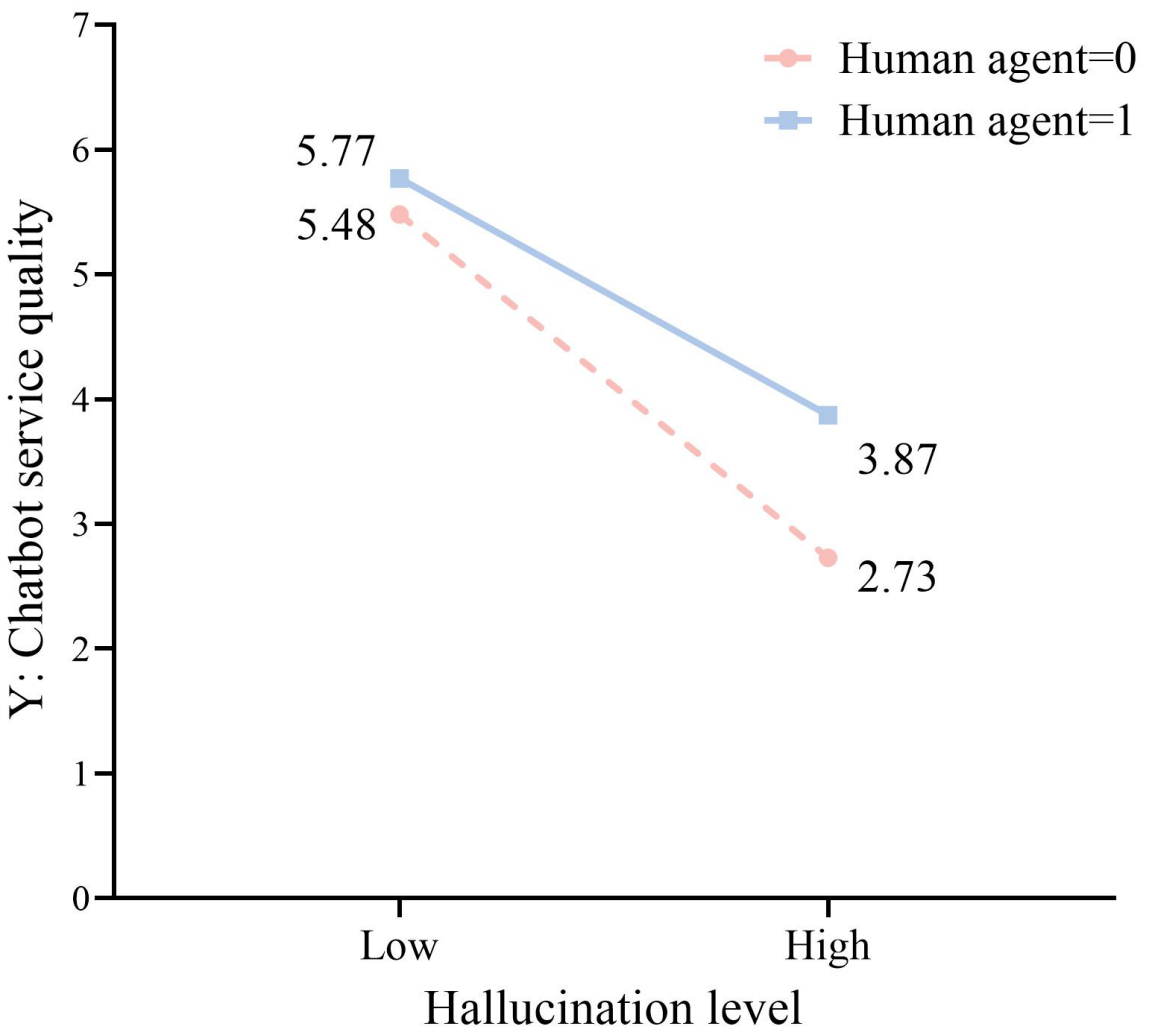
Simple slope analysis.

### 4.3 Study 2

Study 2 aimed to investigate the internal psychological mechanism through which AI hallucination affects the chatbot service quality. A model was developed to test the mediating effects of cognitive dissonance and technical frustration. Building on this, the study further explored the boundary conditions of this mechanism, tested whether the human agent could negatively moderate the impact of AI hallucination on these psychological variables. Regarding the experimental procedures, Study 2 generally followed the experimental design and stimulus materials from Study 1. Expanding on the scope of Study 1, Study 2 incorporated measures of cognitive dissonance and technical frustration into the test scale. Specifically, participants completed the questionnaire immediately following the simulated interaction to capture their real-time psychological responses and subsequent service evaluations while minimizing potential recall decay. For data collection, 380 samples were recruited. Applying the same criteria as in Study 1, 16 invalid responses were excluded, resulting in 364 valid responses after screening. The excluded cases were minimally and evenly distributed across conditions (N = 3 to 5 per group), showing no selective group attrition. Table 4 details the demographic distribution of this sample.

**Table 4:** Demographic distribution of Study 2.

Measurement items	Category	Number of participants	Percentage
Gender	Male	192	52.7%
	Female	172	47.3%
Age	21–30 years	157	43.1%
	31–40 years	118	32.4%
	41–50 years	68	18.7%
	Over 50 years	21	5.8%
Education	High School	30	8.2%
	Undergraduate degree	222	61.0%
	Master’s degree	103	28.3%
	Doctoral degree	9	2.5%
Monthly income	Below ¥3,000	74	20.3%
	¥3,000–¥5,000	149	40.9%
	¥5,000–¥10,000	112	30.8%
	Over ¥10,000	29	8.0%

In this study, SPSS was used for reliability and validity analyses. As shown in Table 5, the Kaiser-Meyer-Olkin (KMO) value is significantly higher than the critical threshold of 0.7, and Bartlett’s test of sphericity reached statistical significance, indicating that the sample data is suitable for factor analysis. In the reliability analysis, both Cronbach’s alpha coefficients and composite reliability (CR) for each variable exceeded 0.7, suggesting ideal internal consistency of the scale. In the validity test, the standardized factor loadings for all measurement items were greater than 0.7, and the average variance extracted (AVE) for each construct surpassed 0.5. These results confirm that the measurement items accurately represent their corresponding latent variables, and that the scale demonstrates good convergent validity. In addition to convergent validity, the Heterotrait-Monotrait (HTMT) ratio was used to evaluate the discriminant validity. As presented in Table 6, all pairwise HTMT values ranged between 0.010 and 0.826, strictly falling below the conservative threshold of 0.85, confirming satisfactory discriminant validity across all latent constructs.

**Table 5:** Reliability and validity analysis.

Indicator		Value
KMO	0.957
Bartlett test of Sphericity	3857.841
df	105
*p*-value	0.000

Factors	Items	Factor loading	Crobach’s alpha	CR	AVE
AI hallucination	Hal_1	0.844	0.90	0.93	0.73
	Hal_2	0.870			
	Hal_3	0.871			
	Hal_4	0.832			
	Hal_5	0.839			
Cognitive dissonance	Cog_1	0.884	0.86	0.92	0.78
	Cog_2	0.884			
	Cog_3	0.890			
Technical frustration	Tec_1	0.879	0.90	0.93	0.78
	Tec_2	0.857			
	Tec_3	0.896			
	Tec_4	0.898			
Chatbot service quality	Ser_1	0.890	0.87	0.91	0.79
	Ser_2	0.894			
	Ser_3	0.882			

**Table 6:** Discriminant validity analysis (HTMT ratio).

Factors	Cognitive dissonance	AI Hallucination	Human agent	Chatbot service quality	Technical frustration	Human agent × AI hallucination
Cognitive dissonance	**–**					
AI Hallucination	0.741	–				
Human agent	0.061	0.044	–			
Chatbot service quality	0.798	0.679	0.232	–		
Technical frustration	0.763	0.742	0.096	0.826	–	
Human agent × AI hallucination	0.500	0.762	0.010	0.337	0.459	–

Figure 6 presents a heatmap illustrating the correlations among the variables in this study. Correlation analysis revealed significant positive associations between AI hallucination and both cognitive dissonance (*r* = 0.65, *p* < 0.01) and technical frustration (*r* = 0.67, *p* < 0.01). These results provide preliminary empirical support for the premise that AI hallucination leads to negative psychological responses in users. Correspondingly, AI hallucination (*r* = −0.60, *p* < 0.01), cognitive dissonance (*r* = −0.69, *p* < 0.01), and technical frustration (*r* = −0.73, *p* < 0.01) were all significantly negatively correlated with chatbot service quality, underscoring the detrimental impact of these negative factors on service evaluations. To further rule out multicollinearity among the variables, the variance inflation factor (VIF) was employed for collinearity diagnosis. The VIF values for all predictor variables were below 5, indicating the absence of serious multicollinearity problems.

**Figure 6:**
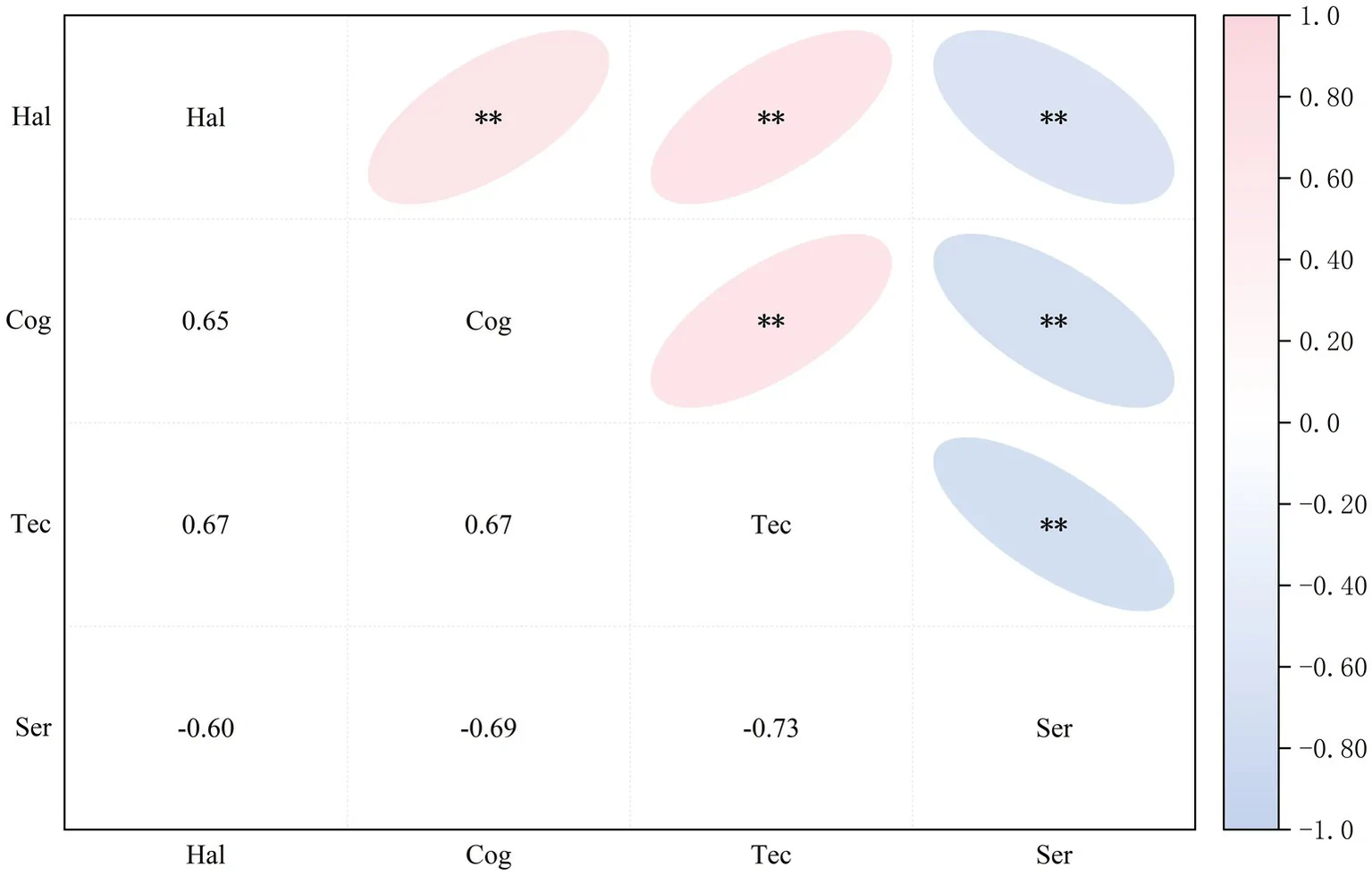
Correlation heatmap.

Following the experimental procedure of Study 1, this study also conducted a manipulation check for AI hallucination. Results from the one-way analysis of variance (ANOVA) revealed a significant difference in AI hallucination between the high-level and low-level groups (*F* (1,362) = 679.89, *p* < 0.001). The mean comparison indicated that the participants’ perception (*M_high_* = 5.37, *SD* = 1.10) was significantly higher than that of the low hallucination group (*M_low_* = 2.54, *SD* = 0.97), confirming the successful manipulation of AI hallucination in Study 2.

After controlling for relevant covariates, this study used an analysis of covariance (ANCOVA) to test for the significance of the main effects and interaction effects of the independent variables. As shown in Table 7, the main effects of AI hallucination on cognitive dissonance (*F* (1,357) = 624.98, *p* < 0.001), technical frustration (*F* (1, 357) = 787.25, *p* < 0.001), and service quality (*F* (1,357) = 563.40, *p* < 0.001) were all significant. For the interaction effects, the interaction term between the human agent and AI hallucination had a significant impact on technical frustration (*F* (1,357) = 9.38, *p* < 0.01) and chatbot service quality (*F* (1,357) = 33.49, *p* < 0.001). However, this interaction term did not significantly affect cognitive dissonance (*F* (1,357) = 0.16, *p* > 0.05). This finding suggests that cognitive dissonance, as an immediate cognitive conflict, is highly stable and difficult to moderate through external compensatory services. Additionally, the robustness test using the full randomized sample (N = 380) confirmed that the main effects and observed moderating effects remained statistically significant, verifying the stability of the empirical results.

**Table 7:** Covariance analysis results (Study 2).

Factors	Cognitive dissonance		Technical frustration		Chatbot service quality	
	*F*	*p*-value	*F*	*p*-value	*F*	*p*-value
AI hallucination	624.975	0.000	787.253	0.000	563.398	0.000
Human agent	3.029	0.083	9.112	0.003	44.443	0.000
AI hallucination × Human agent	0.155	0.694	9.382	0.002	33.489	0.000
Covariate: gender	0.053	0.833	1.224	0.269	0.336	0.562
Covariate: age	0.002	0.961	0.829	0.363	0.100	0.752
Covariate: product shopping experience	1.096	0.296	1.920	0.167	5.975	0.015

To provide evidence consistent with a mediating mechanism through which AI hallucination is linked to service quality, this study utilized SmartPLS 4.0 software to construct a Partial Least Squares Structural Equation Model (PLS-SEM). A Bootstrapping procedure with 5,000 resamples was employed to test the significance of all hypothesized pathways. Figure 7 and Table 8 summarize the structural path estimates, effect sizes, and 95% bias-corrected confidence intervals. Path analysis revealed that AI hallucination was positively associated with users’ cognitive dissonance (*β* = 0.671, *t* = 13.319, *p* < 0.001) and technical frustration (*β* = 0.749, *t* = 16.232, *p* < 0.001), providing support for hypotheses H3 and H4. In addition, the increase in cognitive dissonance (*β* = −0.328, *t* = 7.443, *p* < 0.001) and technical frustration (*β* = −0.410, *t* = 9.100, *p* < 0.001) reduced users’ evaluations of the chatbot service quality. The results of the mediating effect test indicated that AI hallucination exerts an indirect impact on service quality through cognitive dissonance (*β* = −0.220, *t* = 6.736, *p* < 0.001) and technical frustration (*β* = −0.307, *t* = 7.906, *p* < 0.001), thereby supporting hypotheses H5 and H6. After accounting for the mediating variables, the direct effect of AI hallucination on service quality remained significant (*β* = −0.262, *t* = 4.791, *p* < 0.001), indicating that cognitive dissonance and technical frustration play a partial mediating role in the model. The variance accounted for (VAF) was 66.8%, which represents the proportion of the total effect accounted for by the two indirect pathways, and empirically supports the indirect associations between AI hallucination and chatbot service quality. This finding reveals that the harm caused by AI hallucination is not only reflected in the objective deviation of information transmission, but also lies in its weakening of users’ overall perception of the human-computer interaction process through the dual pathway of “cognition-emotion.”

**Figure 7:**
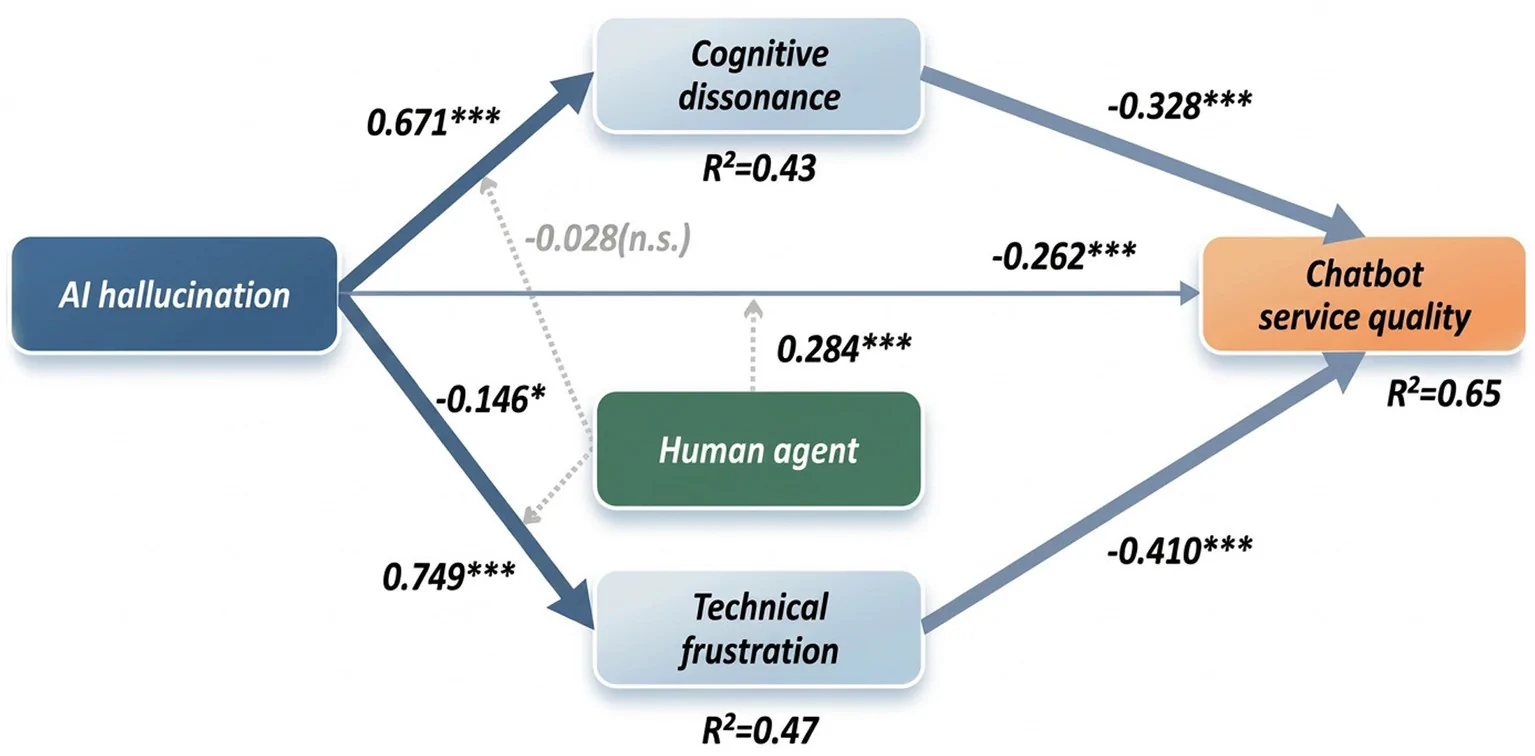
PLS-structural equation model.

**Table 8:** Structural model path estimates, effect sizes, and confidence intervals.

Path	*β*	*SE*	*t*	*p*-value	Effect size (*f*^2^)	Confidence intervals	Significant or not
Direct effects							
Hal → Cog	0.671	0.050	13.319	0.000	0.378	[0.561,0.762]	Significant
Hal → Tec	0.749	0.046	16.232	0.000	0.501	[0.656,0.836]	Significant
Hal → Ser	−0.262	0.055	4.791	0.000	0.058	[−0.369,–0.156]	Significant
Cog→Ser	−0.328	0.044	7.443	0.000	0.146	[−0.412,–0.240]	Significant
Tec → Ser	−0.410	0.045	9.100	0.000	0.215	[−0.501,–0.321]	Significant
Moderating effects							
Human × Hal → Cog	−0.028	0.073	0.376	0.707	0.000	[−0.169,0.117]	Not Significant
Human × Hal → Tec	−0.146	0.068	2.161	0.031	0.010	[−0.280,–0.018]	Significant
Human × Hal → Ser	0.284	0.062	4.594	0.000	0.058	[0.164,0.410]	Significant
Mediating effects							
Hal → Cog → Ser	−0.220	0.033	6.736	0.000	–	[−0.287,–0.159]	Significant
Hal → Tec → Ser	−0.307	0.039	7.906	0.000	–	[−0.389,–0.233]	Significant

It is worth noting that the moderating effect of the human agent based service recovery exhibits heterogeneity across different pathways. Moderating path analysis revealed that the human agent service recovery significantly mitigated the negative impact of AI hallucination on technical frustration (*β* = −0.146, *t* = 2.161, *p* < 0.05) and effectively improved chatbot service quality (*β* = 0.284, *t* = 4.594, *p* < 0.001). However, it showed limited effectiveness in moderating the cognitive dissonance path (*β* = −0.028, *t* = 0.376, *p* > 0.05). As a negative emotional state triggered by system failure, technical frustration exhibits strong plasticity. Real time intervention by a human agent can provide explanations and remedies, thus achieving effective emotional repair. In contrast, cognitive dissonance reflects individuals’ deep-seated psychological defense mechanisms. Once AI hallucination causes a collapse in perceived system authority and form a negative cognitive anchor, it becomes extremely difficult to reshape the user’s damaged cognitive structure in the short term.

## 5 Conclusion

### 5.1 Discussion

Across both scenario-based experiments, participants in the high-hallucination condition evaluated chatbot service quality significantly lower than those in the low-hallucination condition. This finding aligns with research indicating that inaccurate AI generated information may undermine consumer evaluations. For example, Kim et al. (2025) confirmed that when ChatGPT provided travel recommendations without factual errors, users rated their accuracy and credibility more positively. Meanwhile, Christensen et al. (2025) observed that many consumers still chose AI generated travel itineraries containing errors because they perceived the AI provided information as more impartial and personalized. This pattern suggests that the adoption of AI-generated solutions is heavily contingent upon consumers’ prior trust and appraisals of AI as a distinct information channel. In the product consultation task of this study, product specifications and promotional terms directly constitute the information consumers need to verify. The lower service quality ratings may reflect consumers’ perception that the chatbot failed to provide reliable assistance with these specific issues.

The empirical findings of this study support an indirect association between AI hallucination and perceived chatbot quality through cognitive dissonance. Related studies have explored the consequences of cognitive dissonance from the perspectives of usage behavior and purchasing decisions. Zhou and Zhang (2025) linked information hallucination and cognitive dissonance to the intermittent discontinuance of generative AI. Saranya and Joji Alex (2024) discussed the relationship between consumer confusion, cognitive dissonance, and delayed purchase decisions within a conceptual framework. This study further suggests that cognitive dissonance may also manifest in consumers’ service evaluations of chatbots. Upon detecting erroneous guidance, consumers re-evaluate their positive preconceptions regarding the chatbot. Consequently, lowering perceived service quality serves to align these favorable appraisals with the contradictory reality of receiving inaccurate information.

Furthermore, this study supports the indirect association between AI hallucination and perceived chatbot quality via technical frustration. Based on an analysis of mobile app reviews and customer ratings, Hazarika et al. (2025) identified technical frustration as a primary catalyst driving negative shifts in consumer evaluations. Our study further contextualizes this relationship within a product consultation setting, examining how hallucinatory content leads to degraded service quality ratings through the pathway of frustrating experiences. However, other emotional reactions may also arise when users encounter AI errors. In daily recognition tasks, Jeung and Huang (2023) observed that AI errors stimulated user curiosity, prompting exploratory behaviors to investigate why the failure occurred. This points to the fact that the emotional orientation induced by AI errors depends heavily on the nature of the user’s task and interaction goals. The goal impediment perspective proposed by Bessiere et al. (2006) offers a compelling rationale for interpreting our findings. Technical frustration in this context reflects an affective reaction to obstructed goal pursuit when acquiring actionable product information, an adverse experience that systematically impairs overall evaluations of the focal service encounter.

This study demonstrates that human agent based recovery mitigates the adverse effects of AI hallucination on perceived service quality. These measures weakened the positive relationship between hallucination and technical frustration, but their moderating effect on cognitive dissonance was not statistically significant. To understand these results, it is necessary to focus on the specific assistance provided by human intervention. Van Vaerenbergh and Orsingher (2016) noted that consumers evaluate remedial measures based on the outcomes they receive, the remediation process, and the treatment they are given. This provides a basis for explaining the effects of the intervention in this study. In a product inquiry context, human agent addressed consumers’ immediate information needs by apologizing, correcting product and promotional information, and providing transaction verification guidance. A plausible explanation is that these measures helped consumers resolve their immediate issues, thereby alleviating the frustration caused by the consultation barrier and improving service evaluations.

However, the effectiveness of human intervention is limited by the contextual conditions. Huang et al. (2024) found in a travel booking experiment that when consumers had low situational trust in chatbots, even with the subsequent addition of brief human reassurance, hybrid services combining bots and human agents still scored lower than purely human services on all evaluated metrics. Human involvement alone may not be sufficient to eliminate consumers’ negative judgments of the service. In the context of this study, fact correction helped consumers address the information issues, but they were still aware that the chatbot had previously provided incorrect guidance. Even an explicit acknowledgment of the error in the corrective information may not immediately resolve this cognitive dissonance. Therefore, while this study supports testing the role of human agent based service recovery strategies in mitigating some of the consequences of hallucination, it does not yet allow us to conclude that human recovery is superior to AI recovery providing the same content, or that cognitive dissonance cannot be alleviated through other remediation methods.

### 5.2 Theoretical implications

The theoretical implications of this study can be summarized in the following three points. First, this study extends research on AI hallucination by examining its consequences for perceived service quality in e-commerce chatbot interactions. Previous research has developed hallucination detection and mitigation (Ji et al., 2023), as well as users’ protective behaviors and discontinuance of generative AI use (Lee et al., 2025; Zhou and Zhang, 2025). In online markets, informative product descriptions can reduce consumers’ uncertainty about products (Dimoka et al., 2012). In this context, inaccurate or misleading product information from a chatbot represents a content-related failure in the service encounter. Our findings show that higher levels of hallucination lower consumers’ evaluations of chatbot service quality, extending research on chatbot service failures and their consequences for customers.

Second, this study contributes to understanding how AI hallucination relates to service quality evaluations by examining two parallel mediating pathways of cognitive dissonance and technical frustration. Previous research has explored the consequences of AI hallucination from multiple theoretical perspectives. For instance, protection motivation theory suggests that hallucination, as a threat signal, prompts users to verify information (Lee et al., 2025). The theory of technology acceptance posits that an increase in the severity of AI hallucinations weakens users’ attitudes toward AI, thereby reducing their willingness to continue using it (Christensen et al., 2025). Building on these perspectives, the present study focuses on cognitive and emotional responses within a specific service encounter. For cognitive dissonance theory, the findings extend its application to consumers’ service quality evaluations, suggesting that discomfort associated with perceived inaccurate guidance may be reflected in judgments of the assistance received. For frustration theory, the findings extend the application of the goal-obstruction perspective to problems with chatbot response content. Inaccurate information may hinder consumers’ efforts to obtain useful product guidance, even when the system provides a response. The indirect association through technical frustration links this emotional experience to service quality evaluations. These findings offer contextual evidence for the application of both theories to e-commerce consultations.

Third, this study offers a novel theoretical lens for advancing human-AI collaboration. Existing research has largely focused on the internal governance of algorithms and technical level calibration, often treating humans and AI as isolated service providers. In human-machine interaction, the role of humans is not limited to that of passive service recipients, as they can also act as active collaborators, providing support and assurance throughout the interaction process. This study highlights the importance of human agent based recovery strategies in mitigating the negative effects of AI hallucination, providing a theoretical foundation for human-AI collaborative interactions in the e-commerce domain.

### 5.3 Practical implications

This study offers actionable guidance for platform administrators to manage conversational AI hallucinations and optimize service recovery mechanisms. Management must strengthen source-level governance of AI hallucinations, particularly concerning core information that drives purchase decisions. Since the generation of hallucinated information can lead to a decline in service quality, filtering out errors before information is transmitted is essential for protecting the service’s reputation. Implementing strict knowledge base matching and review mechanisms serves as the benchmark for ensuring the accuracy and reliability of critical fulfillment information.

In addition, companies should establish agile human-machine collaborative service remediation mechanisms to standardize the smooth transition from intelligent interactions to human customer service. Timely human intervention helps ensure the smooth progression of inquiries and transactions. To operationalize this, platforms should leverage conversational monitoring algorithms to detect real-time indicators of user interactional bottlenecks and affective frustration, subsequently deploying a tiered incident-response workflow. Upon identifying these risk signals, the system should promptly route the session to human representatives for targeted factual rectifications and emotional reassurance, thereby arresting the detrimental fallout of hallucinatory outputs on consumer shopping experiences.

### 5.4 Limitations and future research

This study has several limitations that suggest directions for future research. The experiments used a scripted chatbot whose product responses were pre-written rather than generated by a live LLM. The recovery message was also pre-written and presented as coming from a human customer service agent. These choices allowed us to control the information shown to the participants, but they did not capture variation in a live model’s responses or the responsiveness of a real human agent. The findings therefore concern participants’ reactions to controlled service scenarios. Their generalizability to deployed LLM chatbots and real human handoffs remains to be established. Future studies could examine these relationships in live interactions while documenting the response errors and recovery content that participants actually encounter.

Regarding the operationalization of hallucination, the experiments compared high- and low-hallucination conditions without an error-free control group. The low-hallucination script contained two errors, whereas the high-hallucination script contained six. Although the manipulation check confirmed a significant difference in perceived AI hallucination between the conditions, participants’ responses may also have been influenced by error content and type, alongside error frequency. Future studies could include an error-free baseline and vary error frequency while holding error type and severity as constant as possible.

The experiments focused on a single product category, namely rice cooker. Consumers’ information needs and the consequences of inaccurate advice may differ across product categories. Future studies could extend the investigation to a broader range of product categories to assess the generalizability of the findings. The consultation was limited to three predefined tasks, and participants were instructed to complete task reading and chatbot interaction within five minutes. The findings therefore concern immediate responses to a brief consultation and do not establish how service evaluations develop through longer or repeated interactions. Future studies could record interaction duration and examine changes in consumers’ responses across multiple service encounters. The study relied on self-report measures, which may be subject to response biases and common method variance. Future research could complement these measures with behavioral data and collect assessments at different time points.

Finally, although this study controlled for product shopping experience and demographics, participants’ prior familiarity with AI chatbots was not measured. Prior experience with and knowledge of conversational AI may influence consumers’ tolerance of errors and their reactions to service failures. Future studies could measure prior chatbot use and AI literacy to examine their roles in shaping cognitive dissonance, technical frustration, and service quality evaluations.

## Data Availability

The raw data supporting the conclusions of this article will be made available by the authors, without undue reservation.

## References

[ref1] Al-Shaikh M. S. Al-Mousa M. R. Al-Ababneh H. A. Ali M. W. Alkaawneh S. M. Barhoush F. M. S. (2024). The challenges of using generative AI applications in electronic commerce 2024 25th International Arab Conference on Information Technology (ACIT) IEEE 1–5

[ref2] Athaluri S. A. Manthena S. V. Kesapragada V. K. M. Yarlagadda V. Dave T. Duddumpudi R. T. S. (2023). Exploring the boundaries of reality: investigating the phenomenon of artificial intelligence hallucination in scientific writing through ChatGPT references. Cureus 15. doi: 10.7759/cureus.37432, 37182055 PMC10173677

[ref3] Bessiere K. Newhagen J. E. Robinson J. P. Shneiderman B. (2006). A model for computer frustration: the role of instrumental and dispositional factors on incident, session, and post-session frustration and mood. Comput. Hum. Behav. 22, 941–961. doi: 10.1016/j.chb.2004.03.015

[ref4] Castillo D. Canhoto A. I. Said E. (2021). The dark side of AI-powered service interactions: exploring the process of co-destruction from the customer perspective. Serv. Ind. J. 41, 900–925. doi: 10.1080/02642069.2020.1787993

[ref5] Ceaparu I. Lazar J. Bessiere K. Robinson J. Shneiderman B. (2004). Determining causes and severity of end-user frustration. Int. J. Hum. Comput. Interact. 17, 333–356.

[ref6] Chakraborty N. Ornik M. Driggs-Campbell K. (2025). Hallucination detection in foundation models for decision-making: a flexible definition and review of the state of the art. ACM Comput. Surv. 57, 1–35.

[ref7] Chen Q. Gong Y. Lu Y. Tang J. (2022). Classifying and measuring the service quality of AI chatbot in frontline service. J. Bus. Res. 145, 552–568. doi: 10.1016/j.jbusres.2022.02.088

[ref8] Chhabra S. Kaushal V. Girija S. (2025). Determining the causes of user frustration in the case of conversational chatbots. Behav. Inf. Technol. 44, 1557–1575. doi: 10.1080/0144929x.2024.2362956

[ref9] Chiang A.-H. Trimi S. Lo Y.-J. (2022). Emotion and service quality of anthropomorphic robots. Technol. Forecast. Soc. Change 177:121550. doi: 10.1016/j.techfore.2022.121550

[ref10] Choudhary V. Marchetti A. Shrestha Y. R. Puranam P. (2025). Human-AI ensembles: when can they work? J. Manag. 51, 536–569. doi: 10.1177/01492063231194968

[ref11] Christensen J. Hansen J. M. Wilson P. (2025). Understanding the role and impact of generative artificial intelligence (AI) hallucination within consumers’ tourism decision-making processes. Curr. Issues Tour. 28, 545–560. doi: 10.1080/13683500.2023.2300032

[ref12] Chugh P. Jain V. (2024). Artificial intelligence (AI) empowerment in e-commerce: a bibliometric voyage. NMIMS Manag. Rev. 32, 159–173. doi: 10.1177/09711023241303621

[ref13] Cooper J. (2019). Cognitive dissonance: where we’ve been and where we’re going. Int. Rev. Soc. Psychol. 32, 7–7. doi: 10.5334/irsp.277

[ref14] Cronin Jr J. J. Brady M. K. Hult G. T. M. (2000). Assessing the effects of quality, value, and customer satisfaction on consumer behavioral intentions in service environments. J. Retail. 76, 193–218. doi: 10.1016/s0022-4359(00)00028-2

[ref15] Dave T. Athaluri S. A. Singh S. (2023). ChatGPT in medicine: an overview of its applications, advantages, limitations, future prospects, and ethical considerations. Front. Artif. Intell. 6:1169595. doi: 10.3389/frai.2023.1169595, 37215063 PMC10192861

[ref16] Demirgüneş B. K. Avcilar M. Y. (2017). The effect of cognitive dissonance on external information search and consumer complaint responses. Int. J. Bus. Adm. 8, 57–72.

[ref17] Dimoka A. Hong Y. Pavlou P. A. (2012). On product uncertainty in online markets: theory and evidence. MIS Q. 36, 395–426. doi: 10.2307/41703461

[ref18] Dissanayake H. Manta O. Iddagoda A. Palazzo M. (2025). Artificial intelligence and management education: bibliometric analysis. Int. J. Manag. Educ. 23:101222. doi: 10.1016/j.ijme.2025.101222

[ref19] Elazar Y. Kassner N. Ravfogel S. Ravichander A. Hovy E. Schütze H. . (2021). Measuring and improving consistency in pretrained language models. Trans. Assoc. Comput. Linguist. 9, 1012–1031. doi: 10.1162/tacl_a_00410

[ref20] Festinger L. (1957). A Theory of Cognitive Dissonance. Evanston, Illinois, USA: Row, Peterson.

[ref21] Fürst A. Trißler L. Friedrich R. Wirtz J. (2025). Service recovery by AI or human agents: do failure and strategy context matter? J. Serv. Manag. 36, 390–418. doi: 10.1108/josm-04-2024-0190

[ref22] Giboney J. S. Brown S. A. Lowry P. B. Nunamaker J. F. (2015). User acceptance of knowledge-based system recommendations: explanations, arguments, and fit. Decis. Support. Syst. 72, 1–10.

[ref23] Han E. Yin D. Zhang H. (2023). Bots with feelings: should AI agents express positive emotion in customer service? Inf. Syst. Res. 34, 1296–1311. doi: 10.1287/isre.2022.1179, 19642375

[ref24] Hazarika B. Shrivastava U. Hiele T. M. Pham C. (2025). The impact of technology frustration and consumer passion on consumer evaluation shift in case of mobile apps. Acta Psychol. 256:105006. doi: 10.1016/j.actpsy.2025.105006, 40250168

[ref25] He R. Cao J. Tan T. (2025). Generative artificial intelligence: a historical perspective. Natl. Sci. Rev. 12. doi: 10.1093/nsr/nwaf050, 40191253 PMC11970245

[ref26] Hertzum M. Hornbæk K. (2023). Frustration: still a common user experience. ACM Trans. Comput. Hum. Interact. 30, 1–26. doi: 10.1145/3582432

[ref27] Huang D. Markovitch D. G. Stough R. A. (2024). Can chatbot customer service match human service agents on customer satisfaction? An investigation in the role of trust. J. Retail. Consum. Serv. 76:103600. doi: 10.1016/j.jretconser.2023.103600

[ref28] Huang L. Yu W. Ma W. Zhong W. Feng Z. Wang H. . (2025). A survey on hallucination in large language models: principles, taxonomy, challenges, and open questions. ACM Trans. Inf. Syst. 43, 1–55. doi: 10.1145/3703155

[ref29] Jeung J.L. Huang J.Y.-C. (2023). "Correct me if I am wrong: exploring how AI outputs affect user perception and trust" Companion Publication of the 2023 Conference on Computer Supported Cooperative work and social Computing 323–327

[ref30] Ji Z. Lee N. Frieske R. Yu T. Su D. Xu Y. . (2023). Survey of hallucination in natural language generation. ACM Comput. Surv. 55, 1–38. doi: 10.1145/3571730

[ref31] Jia J. Chen L. Zhang L. Xiao M. Wu C. (2025). A study on the factors that influence consumers’ continuance intention to use artificial intelligence chatbots in a pharmaceutical e-commerce context. Electron. Libr. 43, 303–321. doi: 10.1108/el-09-2024-0275

[ref32] Jiang K. Qin M. Li S. (2022). Chatbots in retail: how do they affect the continued use and purchase intentions of Chinese consumers? J. Consum. Behav. 21, 756–772. doi: 10.1002/cb.2034

[ref33] Jin L. Shen Z. Alhur A. A. Naeem S. B. (2025). Exploring the determinants and effects of artificial intelligence (AI) hallucination exposure on generative AI adoption in healthcare. Inf. Dev.:02666669251340954. doi: 10.1177/02666669251340954

[ref34] Kamoi R. Zhang Y. Zhang N. Han J. Zhang R. (2024). When can LLMs actually correct their own mistakes? A critical survey of self-correction of LLMs. Trans. Assoc. Comput. Linguist. 12, 1417–1440. doi: 10.1162/tacl_a_00713

[ref35] Kim J. H. Kim J. Park J. Kim C. Jhang J. King B. (2025). When ChatGPT gives incorrect answers: the impact of inaccurate information by generative AI on tourism decision-making. J. Travel Res. 64, 51–73. doi: 10.1177/00472875231212996

[ref36] Kshetri N. (2024). Generative artificial intelligence and e-commerce. Computer 57, 125–128. doi: 10.1109/mc.2023.3340772

[ref37] Lee Y. (2025). Developing a computer-based tutor utilizing generative artificial intelligence (GAI) and retrieval-augmented generation (RAG). Educ. Inf. Technol. 30, 7841–7862. doi: 10.1007/s10639-024-13129-5

[ref38] Lee C. Kim J. Lim J. S. Shin D. (2025). Generative AI risks and resilience: how users adapt to hallucination and privacy challenges. Telematics Inform. Rep. 19:100221. doi: 10.1016/j.teler.2025.100221

[ref39] Lee G.-G. Latif E. Wu X. Liu N. Zhai X. (2024). Applying large language models and chain-of-thought for automatic scoring. Comp. Educ. 6:100213. doi: 10.1016/j.caeai.2024.100213

[ref40] Levental O. Lev Arey D. Lev A. (2023). Passionate hearts, torn loyalties: navigating the interplay between fandom and romance. Front. Psychol. 14:1240271. doi: 10.3389/fpsyg.2023.1240271, 37881219 PMC10595012

[ref41] Li B. Liu L. Mao W. Qu Y. Chen Y. (2023). Voice artificial intelligence service failure and customer complaint behavior: the mediation effect of customer emotion. Electron. Commer. Res. Appl. 59:101261. doi: 10.1016/j.elerap.2023.101261

[ref42] Lim J. S. Shin D. Lee C. Kim J. Zhang J. (2025). The role of user empowerment, AI hallucination, and privacy concerns in continued use and premium subscription intentions: an extended technology acceptance model for generative AI. J. Broadcast. Electron. Media 69, 183–199. doi: 10.1080/08838151.2025.2487679

[ref43] Lincecum L. (2000). The Effects of Software Disruption on goal Commitment, task Self-Efficacy, Computer Self-Efficacy, and test Performance in a Computer-based Instructional task. Lubbock, Texas, USA: Texas Tech University.

[ref44] Lv X. Yang Y. Qin D. Cao X. Xu H. (2022). Artificial intelligence service recovery: the role of empathic response in hospitality customers’ continuous usage intention. Comput. Hum. Behav. 126:106993. doi: 10.1016/j.chb.2021.106993

[ref45] Macpherson F. Platchias D. (2013). Hallucination: Philosophy and Psychology. Cambridge, Massachusetts, USA: MIT Press.

[ref46] Manakul P. Liusie A. Gales M. (2023). Selfcheckgpt: zero-resource black-box hallucination detection for generative large language models Proceedings of the 2023 Conference on Empirical Methods in natural Language Processing 9004–9017

[ref47] Marikyan D. Papagiannidis S. Alamanos E. (2023). Cognitive dissonance in technology adoption: a study of smart home users. Inf. Syst. Front. 25, 1101–1123. doi: 10.1007/s10796-020-10042-3, 32837263 PMC7381864

[ref48] Markovitch D. G. Stough R. A. Huang D. (2024). Consumer reactions to chatbot versus human service: an investigation in the role of outcome valence and perceived empathy. J. Retail. Consum. Serv. 79:103847. doi: 10.1016/j.jretconser.2024.103847

[ref49] Martinez Montes C. Khojah R. (2025). Emotional strain and frustration in LLM interactions in software engineering Proceedings of the 29th International Conference on Evaluation and Assessment in Software Engineering 193–204

[ref50] Mimoun M. S. B. Poncin I. Garnier M. (2012). Case study—embodied virtual agents: an analysis on reasons for failure. J. Retail. Consum. Serv. 19, 605–612.

[ref51] Ozuem W. Ranfagni S. Willis M. Salvietti G. Howell K. (2024). Exploring the relationship between chatbots, service failure recovery and customer loyalty: a frustration–aggression perspective. Psychol. Mark. 41, 2253–2273. doi: 10.1002/mar.22051

[ref52] Park I. Cho J. Rao H. R. (2015). The dynamics of pre-and post-purchase service and consumer evaluation of online retailers: a comparative analysis of dissonance and disconfirmation models. Decis. Sci. 46, 1109–1140.

[ref53] Reich T. Teeny J. D. (2025). Does artificial intelligence cause artificial confidence? Generative artificial intelligence as an emerging social referent. J. Pers. Soc. Psychol. 130, 452–464. doi: 10.1037/pspa0000450 40310217

[ref54] Rivas S. Albertos A. (2023). Potential connection between positive frustration in family leisure time and the promotion of adolescent autonomy. Front. Psychol. 14:1258748. doi: 10.3389/fpsyg.2023.1258748, 37799524 PMC10547906

[ref9001] Rossner A. Ambach M. Pagel S. (2025). “Navigating misinformation: understanding user frustration in AI-driven chatbot interactions”, in International conference on human-computer interaction. (Cham: Springer Nature Switzerland), 263–281.

[ref55] Rungtusanatham M. Wallin C. Eckerd S. (2011). The vignette in a scenario-based role-playing experiment. J. Supply Chain Manag. 47, 9–16. doi: 10.1111/j.1745-493x.2011.03232.x

[ref56] Samy T. M. Asham B. I. Slim S. O. Abohany A. A. (2025). Revolutionizing online shopping with FITMI: a realistic virtual try-on solution. Neural Comput. & Applic. 37, 6125–6144. doi: 10.1007/s00521-024-10843-6

[ref57] Saranya P. Joji Alex N. (2024). Consumer confusion on cognitive dissonance: a conceptual framework. NMIMS Manag. Rev. 32, 14–20.

[ref58] Sharun K. Banu S. A. Pawde A. M. Kumar R. Akash S. Dhama K. . (2023). ChatGPT and artificial hallucinations in stem cell research: assessing the accuracy of generated references–a preliminary study. Ann. Med. Surg. 85, 5275–5278. doi: 10.1097/MS9.0000000000001228, 37811040 PMC10553015

[ref59] Silalahi A. D. K. (2025). Frustration dissonance and the paradox of persistence in ChatGPT usage: insights from Indonesia and Taiwan. Int. J. Hum. Comput. Interact. 42, 8546–8577. doi: 10.1080/10447318.2025.2567967

[ref60] Song Y. Cui M. Wan F. Yu Z. Jiang J. (2025). AI hallucination in crisis self-rescue scenarios: the impact on AI service evaluation and the mitigating effect of human expert advice. Int. J. Hum. Comput. Interact. 41, 14419–14439. doi: 10.1080/10447318.2025.2483858

[ref61] Vaghefi I. Qahri-Saremi H. Turel O. (2020). Dealing with social networking site addiction: a cognitive-affective model of discontinuance decisions. Internet Res. 30, 1427–1453. doi: 10.1108/intr-10-2019-0418

[ref62] Van Vaerenbergh Y. Orsingher C. (2016). Service recovery: an integrative framework and research agenda. Acad. Manag. Perspect. 30, 328–346. doi: 10.5465/amp.2014.0143

[ref63] Vedula N. Rokhlenko O. Malmasi S. (2024). "Question suggestion for conversational shopping assistants using product metadata" Proceedings of the 47th International ACM SIGIR Conference on Research and Development in Information Retrieval 2960–2964

[ref64] Wan G. Wu Y. Chen J. Li S. (2025). Reasoning aware self-consistency: leveraging reasoning paths for efficient llm sampling Proceedings of the 2025 Conference of the Nations of the Americas Chapter of the Association for Computational Linguistics: Human Language Technologies (1: Long Papers) 3613–3635

[ref65] Wang Y. Guo R. (2026). The bright side of fictional information: positive impacts of AI hallucination on tourists’ cultural contact. J. Retail. Consum. Serv. 89:104588. doi: 10.1016/j.jretconser.2025.104588

[ref66] Wang R. He Y. Xu J. Zhang H. (2020). Fake news or bad news? Toward an emotion-driven cognitive dissonance model of misinformation diffusion. Asian J. Commun. 30, 317–342.

[ref67] Wang X. Wei J. Schuurmans D. Le Q. Chi E. Narang S. . (2022). Self-consistency improves chain of thought reasoning in language models. arXiv preprint arXiv. 2203.11171

[ref68] Williamson S. M. Prybutok V. (2024). The era of artificial intelligence deception: unraveling the complexities of false realities and emerging threats of misinformation. Information 15:299. doi: 10.3390/info15060299

[ref69] Xiao Y. Chen S. Gallifant J. Bitterman D. Hartvigsen T. Ghassemi M. (2026). Kscope: a framework for characterizing the knowledge status of language models. Adv. Neural Inf. Process. Syst. 38, 114205–114239. doi: 10.52202/085713-3446

[ref70] Yang J. Dong C. Chu S.-C. Rheu M. (2025). Transforming advertising in the age of generative AI: exploring advertising professionals’ perceptions of human-AI value co-creation. Int. J. Advert. 45, 139–170. doi: 10.1080/02650487.2025.2604910

[ref71] Zhang R. W. Liang X. Wu S.-H. (2024). When chatbots fail: exploring user coping following a chatbots-induced service failure. Inf. Technol. People 37, 175–195. doi: 10.1108/itp-08-2023-0745

[ref72] Zhao W. Zhou K. Li J. Tang T. Dong Z. Hou Y. . (2026). A survey of large language models. Front. Comput. Sci. 20:2012627. doi: 10.1007/s11704-026-60308-3

[ref73] Zhou T. Zhang C. (2025). Examining generative AI user intermittent discontinuance from a CAC perspective. Int. J. Hum. Comput. Interact. 41, 6377–6387. doi: 10.1080/10447318.2024.2376370

