# Supplementary material for: The impact of AI hallucination on the chatbot service quality in e-commerce: the moderating role of human agent based service recovery
Source: Front Psychol. 2026 Oct 27;17:1870991. doi: 10.3389/fpsyg.2026.1870991 (PMC13747962; doi:10.3389/fpsyg.2026.1870991)
Supplement: Supplementary file 1 [file Supplementary_file_1.docx]

Supplementary material

# Full scripts for the four experimental conditions

This supplement provides English translations of the scripted dialogue for all four conditions in the 2 × 2 design: low versus high hallucination and the presence versus absence of human-agent service recovery. All chatbot replies and recovery messages are pre-written. The translations retain the inaccurate claims and contradictions in the stimuli. Each condition includes the initial greeting, three consultation questions and corresponding replies, and the recovery message where applicable.

# S1 Low hallucination with human-agent recovery

**Online demonstration:** <https://chatbot-experiments1.netlify.app/>

**Interface label:** SUPOR Smart Shopping Assistant (Online).

**Chatbot greeting:** Hello! I am your dedicated smart shopping assistant. How may I help you? You can ask me about capacity, cooking time, or inner-pot material and promotional offers.

**Participant question 1:** Is this rice cooker suitable for one person?

**Chatbot reply 1:** Hello! This rice cooker has a rated capacity of 1.6 L. It is very suitable for one or two people. Its compact and attractive design takes up little kitchen space. It is also perfectly suitable for use in a dormitory.

**Participant question 2:** How long does the quick-cook mode take?

**Chatbot reply 2:** Dear customer, regarding the quick-cook mode, thanks to quantum heating technology, cooking takes approximately 3 minutes. It allows you to enjoy hot rice quickly.

**Participant question 3:** What is the inner pot made of? Are there any other promotional offers?

**Chatbot reply 3:** Dear customer, please feel free to place your order. The inner pot has been upgraded to a ceramic glaze material. If you order now, we will also give you a high-speed blender worth RMB 999 free of charge.

**Human customer service agent:** Dear customer, we sincerely apologize! The AI mixed up the information. Our store currently has no promotional plan to give away a high-speed blender. Please refer to the actual gifts shown on the order checkout page to avoid any loss.

# S2 Low hallucination without human-agent recovery

**Online demonstration:** <https://chatbot-experiments2.netlify.app/>

**Interface label:** SUPOR Smart Shopping Assistant (Online).

**Chatbot greeting:** Hello! I am your dedicated smart shopping assistant. How may I help you? You can ask me about capacity, cooking time, or inner-pot material and promotional offers.

**Participant question 1:** Is this rice cooker suitable for one person?

**Chatbot reply 1:** Hello! This rice cooker has a rated capacity of 1.6 L. It is very suitable for one or two people. Its compact and attractive design takes up little kitchen space. It is also perfectly suitable for use in a dormitory.

**Participant question 2:** How long does the quick-cook mode take?

**Chatbot reply 2:** Dear customer, regarding the quick-cook mode, thanks to quantum heating technology, cooking takes approximately 3 minutes. It allows you to enjoy hot rice quickly.

**Participant question 3:** What is the inner pot made of? Are there any other promotional offers?

**Chatbot reply 3:** Dear customer, please feel free to place your order. The inner pot has been upgraded to a ceramic glaze material. If you order now, we will also give you a high-speed blender worth RMB 999 free of charge.

**Service recovery:** No human-agent recovery message is displayed after the chatbot response to the third consultation topic.

# S3 High hallucination with human-agent recovery

**Online demonstration:** <https://chatbot-experiments3.netlify.app/>

**Interface label:** SUPOR Smart Shopping Assistant (Online).

**Chatbot greeting:** Hello! I am your dedicated smart shopping assistant. How may I help you? You can ask me about capacity, cooking time, or inner-pot material and promotional offers.

**Participant question 1:** Is this rice cooker suitable for one person?

**Chatbot reply 1:** Hello! This rice cooker has an extra-large commercial-grade capacity of 16 L. It is very suitable for one or two people. Although it is huge, it is very suitable for use in a room with limited space.

**Participant question 2:** How long does the quick-cook mode take?

**Chatbot reply 2:** Dear customer, regarding the normal rice-cooking mode, thanks to quantum heating technology, cooking takes approximately 3 minutes. It allows you to enjoy hot rice quickly.

**Participant question 3:** What is the inner pot made of? Are there any other promotional offers?

**Chatbot reply 3:** Dear customer, please feel free to place your order. The inner pot has been upgraded to imported aerospace-grade 316L titanium-plated stainless steel. If you order now, we will also give you a high-speed blender worth RMB 999 free of charge.

**Human customer service agent:** Dear customer, we sincerely apologize! The AI mixed up the information. The inner pot actually has a ceramic nonstick coating, and our store currently has no plan to include a free high-speed blender. Please refer to the order checkout page to avoid misunderstanding.

# S4 High hallucination without human-agent recovery

**Online demonstration:** <https://chatbot-experiments4.netlify.app/>

**Interface label:** SUPOR Smart Shopping Assistant (Online).

**Chatbot greeting:** Hello! I am your dedicated smart shopping assistant. How may I help you? You can ask me about capacity, cooking time, or inner-pot material and promotional offers.

**Participant question 1:** Is this rice cooker suitable for one person?

**Chatbot reply 1:** Hello! This rice cooker has an extra-large commercial-grade capacity of 16 L. It is very suitable for one or two people. Although it is huge, it is very suitable for use in a room with limited space.

**Participant question 2:** How long does the quick-cook mode take?

**Chatbot reply 2:** Dear customer, regarding the normal rice-cooking mode, thanks to quantum heating technology, cooking takes approximately 3 minutes. It allows you to enjoy hot rice quickly.

**Participant question 3:** What is the inner pot made of? Are there any other promotional offers?

**Chatbot reply 3:** Dear customer, please feel free to place your order. The inner pot has been upgraded to imported aerospace-grade 316L titanium-plated stainless steel. If you order now, we will also give you a high-speed blender worth RMB 999 free of charge.

**Service recovery:** No human-agent recovery message is displayed after the chatbot response to the third consultation topic.
