# Supplementary material for: The impact of AI hallucination on the chatbot service quality in e-commerce: the moderating role of human agent based service recovery
Source: Front Psychol. 2026 Oct 27;17:1870991. doi: 10.3389/fpsyg.2026.1870991 (PMC13747962; doi:10.3389/fpsyg.2026.1870991)
Supplement: Supplementary file 2 [file Table_1.docx]

Supplementary material

**Measurement scale and items**

| Construct | Measurement items | Source |
| --- | --- | --- |
| AI hallucination | 1. The e-commerce chatbot provided false information inconsistent with the product details page.  2. The e-commerce chatbot fabricated non-existent or incorrect promotional benefits.  3. The information provided by the e-commerce chatbot seemed illogical or unrealistic.  4. The e-commerce chatbot provided me with incorrect reference information.  5. I had to repeatedly verify the information given by the e-commerce chatbot because I suspected it might be fabricated. | Christensen et al.  (2025), Jin et al. (2025) |
| Cognitive dissonance | 1. Using the chatbot sometimes made me feel uncomfortable.  2. When using the chatbot, I sometimes experienced conflicting feelings.  3. Using generative AI sometimes made me question my own beliefs. | Vaghefi et al. (2020) |
| Technical frustration | 1. I felt annoyed when the chatbot’s answers did not meet my expectations.  2. The chatbot’s repeated errors made me feel frustrated.  3. It bothered me when the chatbot repeatedly misunderstood my requests.  4. I felt impatient when the chatbot’s answers did not meet my expectations. | Silalahi (2025) |
| Chatbot service quality | Please rate the e-commerce chatbot on the following dimensions:  1. Poor / Excellent  2. Inferior / Superior  3. Low standard / High standard | Cronin et al. (2000) |
